# Peptidoglycan-Targeted [^18^F]3,3,3-Trifluoro-d-alanine Tracer for Imaging Bacterial Infection

**DOI:** 10.1021/jacsau.3c00776

**Published:** 2024-02-26

**Authors:** Alexandre M. Sorlin, Marina López-Álvarez, Jacob Biboy, Joe Gray, Sarah J. Rabbitt, Junaid Ur Rahim, Sang Hee Lee, Kondapa Naidu Bobba, Joseph Blecha, Mathew F.L. Parker, Robert R. Flavell, Joanne Engel, Michael Ohliger, Waldemar Vollmer, David M. Wilson

**Affiliations:** † Department of Radiology, Biomedical Imaging University of California, San Francisco, San Francisco, California 94158, United States; ‡ The Centre for Bacterial Cell Biology, Newcastle University Newcastle, Newcastle upon Tyne NE2 4AX, United Kingdom; § Department of Psychiatry, Renaissance School of Medicine at Stony Brook University, Stony Brook, New York 11794, United States; ∥ UCSF Helen Diller Family Comprehensive Cancer Center, University of California, San Francisco, San Francisco, California 94158, United States; ⊥ Department of Pharmaceutical Chemistry, University of California, San Francisco, San Francisco, California 94158, United States; # Department of Medicine, University of California, San Francisco, San Francisco, California 94158, United States; ∇ Department of Microbiology and Immunology, University of California, San Francisco, San Francisco, California 94158, United States; ○ Department of Radiology, Zuckerberg San Francisco General Hospital, San Francisco, California 94110, United States; ◆ Institute for Molecular Bioscience, The University of Queensland, Brisbane 4072, Australia

**Keywords:** infection imaging, positron emission tomography, peptidoglycan, d-amino acids, radiotracer, metabolism

## Abstract

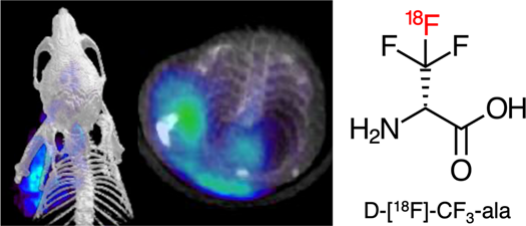

Imaging is increasingly used to detect and monitor bacterial infection. Both anatomic (X-rays, computed tomography, ultrasound, and MRI) and nuclear medicine ([^111^In]-WBC SPECT, [^18^F]FDG PET) techniques are used in clinical practice but lack specificity for the causative microorganisms themselves. To meet this challenge, many groups have developed imaging methods that target pathogen-specific metabolism, including PET tracers integrated into the bacterial cell wall. We have previously reported the d-amino acid derived PET radiotracers d-methyl-[^11^C]-methionine, d-[3-^11^C]-alanine, and d-[3-^11^C]-alanine-d-alanine, which showed robust bacterial accumulation *in vitro* and *in vivo*. Given the clinical importance of radionuclide half-life, in the current study, we developed [^18^F]3,3,3-trifluoro-d-alanine (d-[^18^F]-CF_3_-ala), a fluorine-18 labeled tracer. We tested the hypothesis that d-[^18^F]-CF_3_-ala would be incorporated into bacterial peptidoglycan given its structural similarity to d-alanine itself. NMR analysis showed that the fluorine-19 parent amino acid d-[^19^F]-CF_3_-ala was stable in human and mouse serum. d-[^19^F]-CF_3_-ala was also a poor substrate for d-amino acid oxidase, the enzyme largely responsible for mammalian d-amino acid metabolism and a likely contributor to background signals using d-amino acid derived PET tracers. In addition, d-[^19^F]-CF_3_-ala showed robust incorporation into *Escherichia coli* peptidoglycan, as detected by HPLC/mass spectrometry. Based on these promising results, we developed a radiosynthesis of d-[^18^F]-CF_3_-ala via displacement of a bromo-precursor with [^18^F]fluoride followed by chiral stationary phase HPLC. Unexpectedly, the accumulation of d-[^18^F]-CF_3_-ala by bacteria *in vitro* was highest for Gram-negative pathogens in particular *E. coli*. In a murine model of acute bacterial infection, d-[^18^F]-CF_3_-ala could distinguish live from heat-killed *E. coli*, with low background signals. These results indicate the viability of [^18^F]-modified d-amino acids for infection imaging and indicate that improved specificity for bacterial metabolism can improve tracer performance.

## Introduction

**Figure 1:**
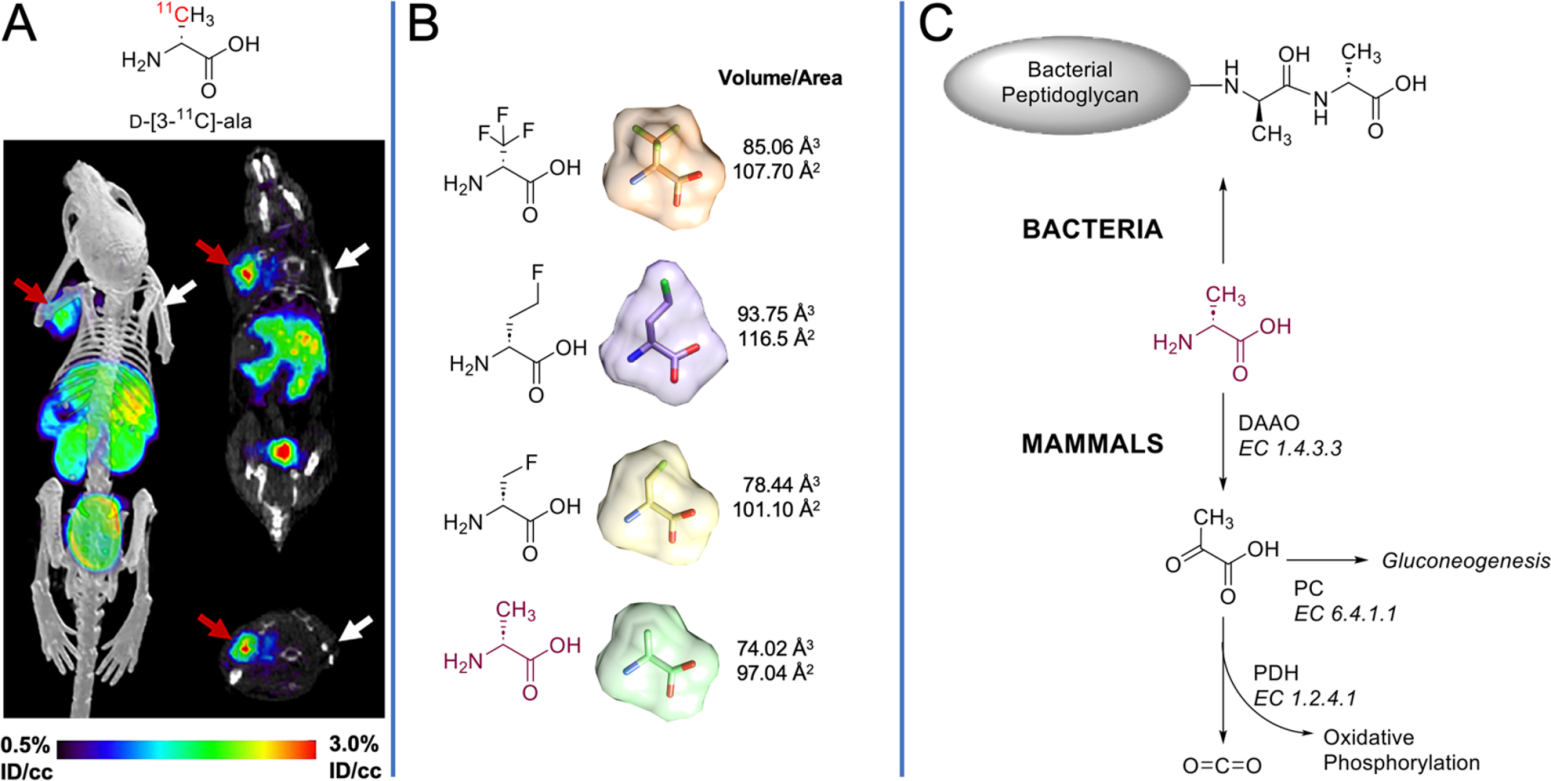
Motivations for pursuing d-[^18^F]-CF_3-_ala as a pathogen-targeted PET radiotracer. (A) Previously reported d-[3-^11^C]-ala tracer in a murine myositis model. The red arrow corresponds to the site of live bacterial inoculation, while the white arrow represents inoculation with 10-fold heat-killed bacteria. The infected site showed robust accumulation with background signals thought to reflect mammalian d-alanine metabolism. Reproduced with permission from Parker et al.^19^ Copyright American Chemical Society. (B) Size comparison of fluorinated analogs of d-alanine. Three-dimensional structures were constructed and minimized in Avogadro modeling software. Minimized structures were visualized in UCSF Chimera, where surface rendering was added, and the volume and surface areas were calculated. (C) Simplified scheme for the metabolism of native d-alanine in an infected animal. In bacteria, d-alanine (and the chemically identical d-[3-^11^C]-ala) are incorporated into bacterial peptidoglycan by the action of LD-TPases and DD-TPases in peptide positions 4 and 5 respectively, resulting in retention in the bacterial cell wall. In mammals, d-alanine is oxidized in mammalian tissues by the FAD-dependent enzyme d-amino acid oxidase (DAAO).^24^ Conversion to [3-^11^C]pyruvate is subsequently expected to label numerous mammalian pathways. The hypothesis of this study was that the mammalian conversion of d-[^18^F]-CF_3_-ala would be suppressed relative to that of native d-alanine.

Commonly used clinical radiotracers including 2-deoxy-2-[^18^F]-fluoro-d-glucose ([^18^F]FDG), [^67^Ga]gallium citrate, and [^111^In]-labeled white blood cells^1^ frequently lack specificity for infection and instead identify the host inflammatory response that accompanies many noninfectious diseases. Recently, several new diagnostic strategies have been developed to target bacteria-specific metabolic pathways, including methods that use labeled metabolites for positron emission tomography (PET). Numerous targets include bacteria-specific sugars and sugar alcohols, substrates for cofactor biosynthesis, and small-molecule iron chelating agents (siderophores).^2−11^ One approach is to radiolabel d-amino acids, which are incorporated efficiently into bacterial peptidoglycan. This strategy has been extensively validated via direct incorporation of fluorescent d-amino acids^12,13^ or functionalization using azide or alkyne-bearing d-amino acids that are subsequently discovered using bioorthogonal chemistry.^14^ The positron-labeled d-amino acid derivatives previously developed for infection have been limited in their ultimate use for human imaging because they employed the relatively short half-life carbon-11 isotope (*t*_1/2_ = 20 min) to label d-[methyl-^11^C]-methionine,^15−17^d-[5-^11^C]-glutamine,^18^d-[3-^11^C]-alanine, and d-[3-^11^C]-alanine-d-alanine.^19,20^ Of these, d-[3-^11^C]-alanine showed the highest uptake into both Gram-negative and Gram-positive bacteria (notably, *Pseudomonas aeruginosa*, *Escherichia coli*, and *Staphylococcus aureus*) and was used to identify bacterial infection in preclinical models of myositis, pneumonia, and discitis-osteomyelitis (Figure 1A).

Based on these preclinical data, d-[3-^11^C]-alanine is a strong candidate for clinical translation but would be difficult to use in acute care settings. The short half-life of carbon-11 is not compatible with facilities lacking an on-site cyclotron, and the logistics of radiosynthesis, quality control, and tracer transport may prohibit the administration of d-[3-^11^C]-alanine to acutely ill patients. The development of fluorine-18 (*t*_1/2_ = 110 min)-labeled d-amino acid-derived PET radiotracers could solve these challenges. Based on published results, d-amino acid derivatives with smaller side chains were incorporated more robustly into bacteria.^21^ These data motivated us to develop fluorine-18 analogs of d-alanine itself that matched the endogenous amino acid in terms of size, charge, and hydrophobicity (Figure 1B). We noted that for several potential radiosynthetic targets, the l-[^18^F]-amino acid derived analogs may be defluorinated *in vivo*,^22,23^ stimulating our pursuit of more stable fluorine-18 radiopharmaceuticals. A second major limitation with d-[3-^11^C]-alanine is that it is metabolized by mammals, potentially producing background PET signals in the host. The biodistribution of d-[3-^11^C]-alanine in noninfected animals indicated high tracer uptake in the liver, lungs, and pancreas, which would limit the detection of bacteria in those locations.^19^ These background signals were likely due to d-amino acid oxidase (DAAO),^24^ an FAD-dependent flavoenzyme that catalyzes the oxidation of d-alanine to pyruvate. This process is remarkably efficient, as it converts hyperpolarized d-[1-^13^C]-alanine to [1-^13^C]-pyruvate in mice within seconds.^25^ Following the conversion of the d-[3-^11^C]-alanine PET tracer to [3-^11^C]-pyruvate, numerous biomolecules in glycolysis, the TCA cycle, and fatty acid biosynthesis would be subsequently labeled (Figure 1C).

These considerations motivated the design of a d-amino acid-derived PET tracer that (1) was fluorine-18 labeled, (2) had high structural homology to d-alanine, (3) was stable *in vivo*, and (4) was incorporated into bacterial peptidoglycan but was not a substrate for mammalian DAAO. A fluorine-18 containing d-alanine derivative appeared feasible, due to the reported promiscuity of unnatural d-amino acid incorporation, especially the apparent tolerance of extracytoplasmic dd- and ld-transeptidases for side-chain modified substrates.^26^ Based on this analysis, [^18^F]3,3,3-trifluoro-d-alanine was an attractive target, given both its similar size to native d-alanine and the relative stability of the [^18^F]trifluoromethyl group versus primary fluorine substrates. The trifluoromethyl group is frequently employed in drugs and drug-like molecules,^27^ and its derivatives can be more stable in aqueous solution versus their *N*-methyl analogs.^28^ While [^18^F]trifluoromethylation chemistry has historically produced PET tracers of low molar activity and radiochemical purity, recent innovations have expanded the radiochemical toolbox.^29^ The general methods of generating [^18^F]trifluoromethyl groups include isotopic exchange,^30^ difluorocarbene-mediated chemistry,^31,32^ electrophilic substitution,^33^ and nucleophilic substitution.^34−36^ Of these, we considered nucleophilic substitution most promising since an appropriate −CF_2_Br precursor could be synthesized from a protected glycine analog.

In this report, we show that [^19^F]3,3,3-trifluoro-d-alanine (d-[^19^F]-CF_3_-ala) is incorporated into bacterial peptidoglycan but is a poor substrate for DAAO, and that the positron-labeled analog d-[^18^F]-CF_3_-ala is accumulated in numerous pathogens *in vitro*. When the tracer was used in a preclinical model of acute *E. coli* infection, it allowed imaging of living bacteria with lower background signals compared to d-[3-^11^C]-alanine.

## Results

### d-alanine Analogue d-[^19^F]-CF_3_-ala Was Serum-Stable, a Poor Substrate for Mammalian d-Amino Acid Oxidase, and Readily Incorporated into *E. coli* Peptidoglycan

**Figure 2:**
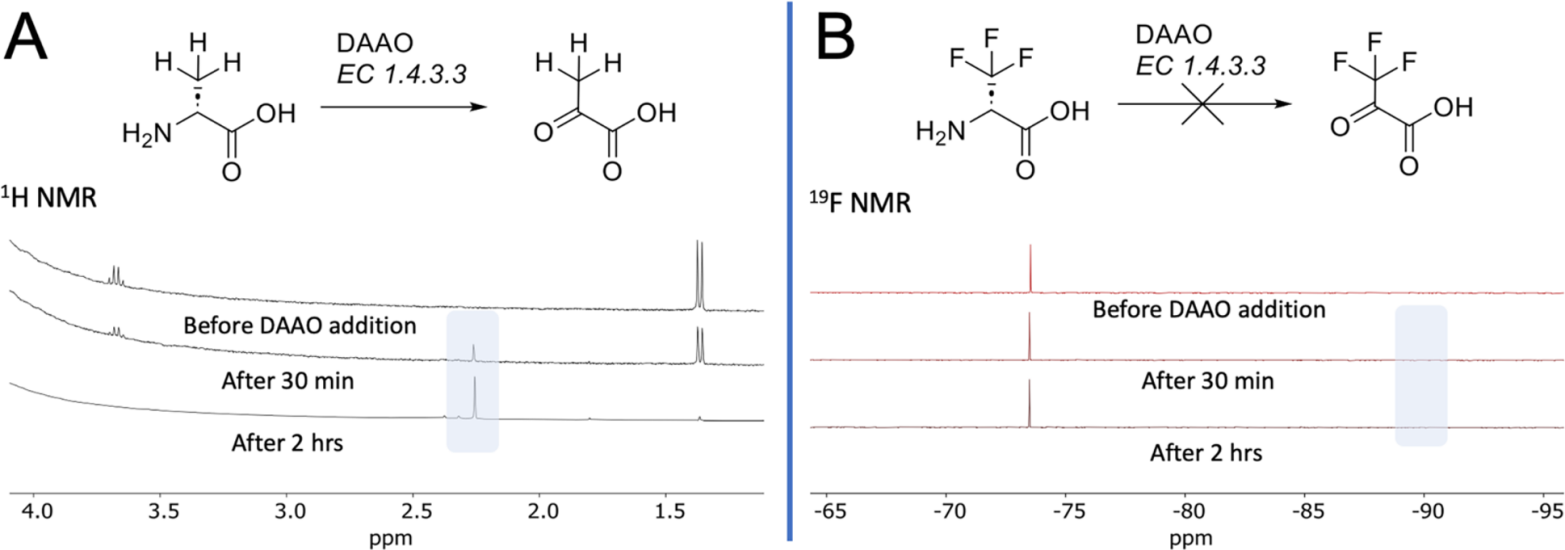
NMR analysis ofd-ala and d-CF_3_-ala specificity for DAAO. (A) d-alanine itself was readily oxidized to pyruvate catalyzed by DAAO, as detected using ^1^H NMR showing evolution of the 3-position singlet. (B) Under identical conditions, the ^19^F-NMR of d-[^19^F]-CF_3_-ala showed no conversion to the fluorinated pyruvate analog (expected location highlighted around −90 ppm).

**Figure 3:**
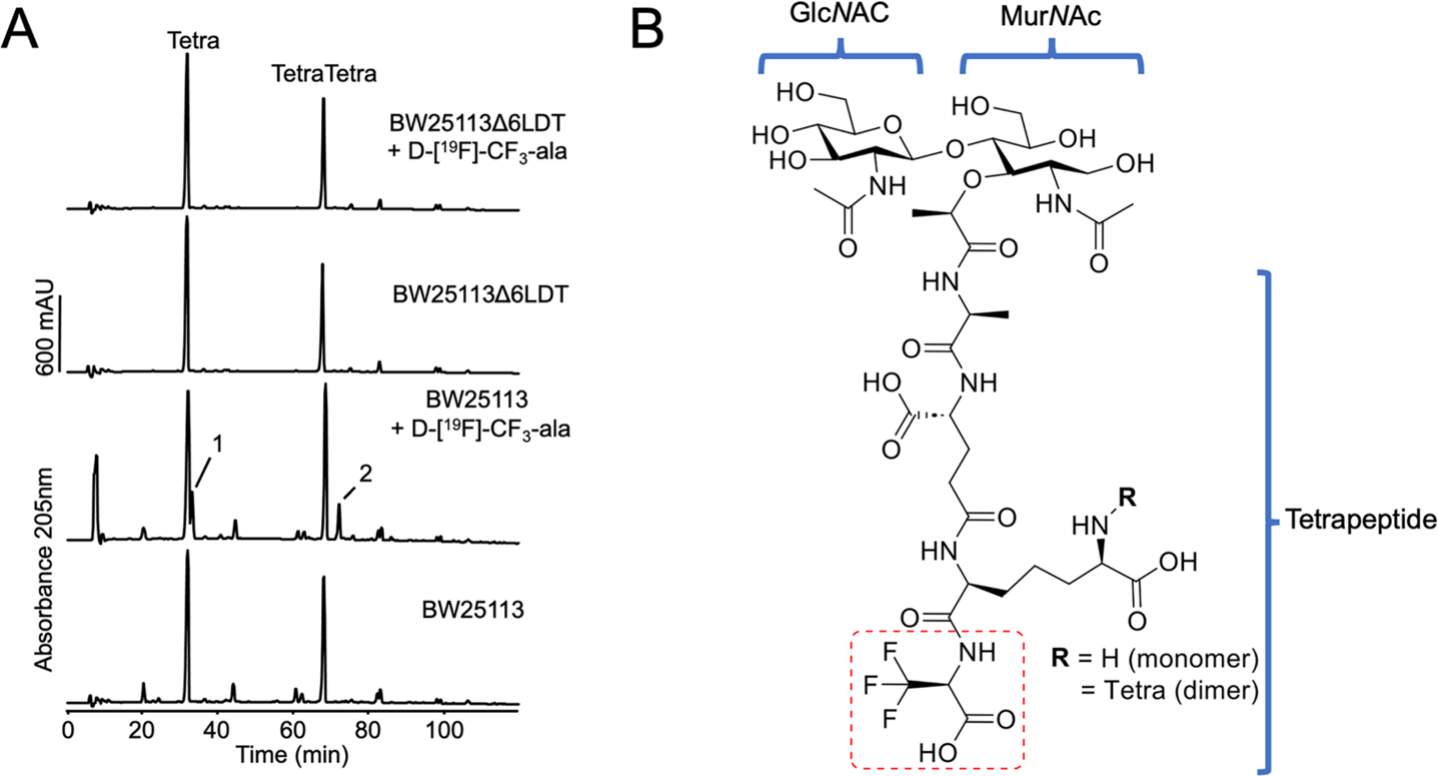
Incorporation of d-[^19^F]-CF_3_-ala into peptidoglycan of *E. coli*. (A) *E. coli* BW25113 (wild-type) and BW25113ΔLDT were grown in the presence or absence of 1 mM d-[^19^F]-CF_3_-ala. Peptidoglycan was isolated and digested with cellosyl to muropeptides, which were reduced with sodium borohydride and separated by HPLC. d-[^19^F]-CF_3_-ala was incorporated into the peptidoglycan of the wild-type, resulting in peaks 1 and 2. MS analysis (Figures S2–S7) confirmed that peaks 1 and 2 are d-[^19^F]-CF_3_-ala modified versions of the major muropeptides, Glc*N*Ac-Mur*N*Ac-l-ala-d-iso-glu-meso-dap-d-ala (Tetra) and Glc*N*Ac-Mur*N*Ac-l-ala-d-iso-glu-meso-dap-(d-ala)-d-ala-meso-dap-d-iso-glu-l-ala-MurNAc-GlcNAc (TetraTetra). Strain BW25113Δ6LDT does not incorporate d-[^19^F]-CF_3_-ala into its PG due to the lack of ld-transpeptidases. (B) Structures of modified muropeptides identified by MS containing d-[^19^F]-CF_3_-ala. Of note Mur*N*Ac is present in its reduced form based on sodium borohydride processing.

To investigate the feasibility of d-[^18^F]-CF_3_-ala as a bacteria-specific PET imaging agent, we first assessed nonradioactive d-[^19^F]-CF_3_-ala behavior via NMR and tested whether it was incorporated into *E. coli* peptidoglycan *in vitro*. Our hypothesis was that d-[^19^F]-CF_3_-ala, due to its structural similarity with the canonical muropeptide C-terminal d-alanine residues, would readily modify peptidoglycan. However, as noted above, two potential pitfalls were the defluorination of d-[^18^F]-CF_3_-ala *in vivo* and metabolism by mammalian DAAO, resulting in the corresponding pyruvate derivative and potentially undesired background signals. We therefore performed several experiments to address whether d-[^19^F]-CF_3-_ala would be defluorinated *in vivo* or metabolized by DAAO. First, we tested the stability of nonradioactive d-[^19^F]-CF_3_-ala in human and mouse serum using ^19^F NMR (Figure S1). There was no degradation observed over 6 h. Second, we evaluated the oxidation of d-[^19^F]-CF_3_-ala by DAAO *in vitro*, comparing its enzymatic conversion to that of d-alanine itself. Incubation of d-alanine with DAAO and catalase for 2 h resulted in the expected conversion to pyruvate (^1^H NMR, Figure 2a), whereas under identical conditions, d-[^19^F]-CF_3_-ala remained intact (^19^F NMR, Figure 2b). Finally, we sought direct evidence for d-[^19^F]-CF_3_-ala incorporation into the bacterial peptidoglycan. Cultures of wild-type *E. coli* and an *E. coli* strain lacking ld-TPases (unable to accommodate exogenous d-amino acids) were incubated with d-[^19^F]-CF_3_-ala. Peptidoglycan was subsequently isolated and digested with muramidase, and the resulting muropeptides were analyzed using HPLC and mass spectroscopy (Figure 3 and Figures S2–S7). New peaks were observed in the monomer and dimer regions of the chromatogram for the d-[^19^F]-CF_3_-ala treated wild-type strain but not for the d-[^19^F]-CF_3_-ala-treated strain lacking ld-TPase. Mass spectroscopy confirmed that the new peaks represented the peptidoglycan monomer with a tetrapeptide containing d-[^19^F]-CF_3_-ala instead of d-alanine, and the peptidoglycan dimer (with cross-linked tetra–tetrapeptide) containing d-[^19^F]-CF_3_-ala instead of d-alanine in the acceptor peptide (Figures S2–S7). As expected, there were few pentapeptides in both profiles due to their efficient conversion into tetrapeptides by the cellular peptidoglycan dd-carboxypeptidases, which are also capable of removing incorporated fluorescent d-Ala-modified pentapeptides.^26^ Overall, these results indicated that d-[^19^F]-CF_3_-ala was a substrate for bacterial ld-transpeptidases. Importantly, d-[^19^F]-CF_3_-ala was not metabolized by mammalian enzyme DAAO.

### Radiosynthesis and Purification of d-[^18^F]-CF_3_-ala

**Figure 4:**
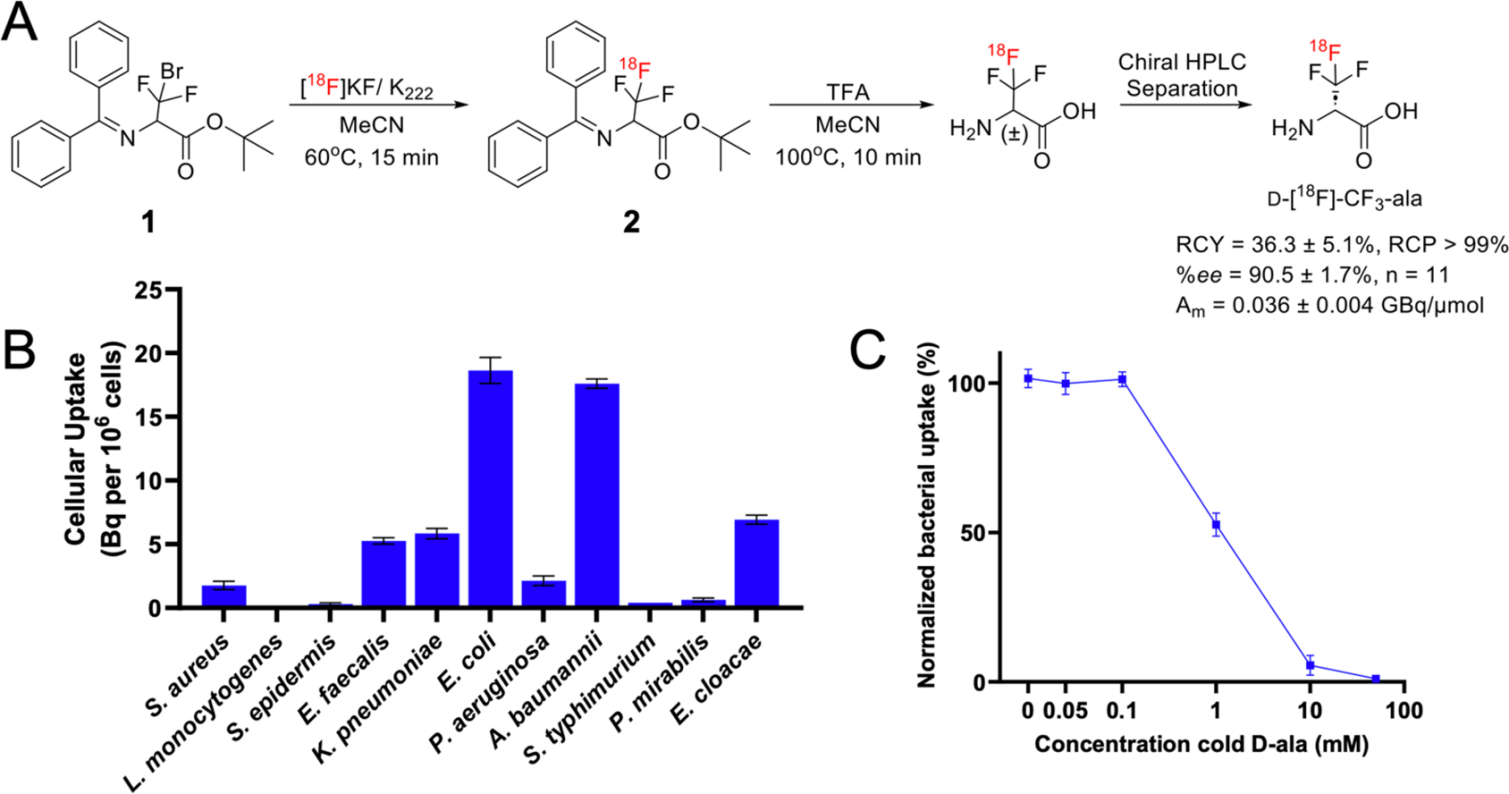
Radiosynthesis and *in vitro* evaluation of d-[^18^F]-CF_3_-ala. (A) d-[^18^F]-CF_3_-ala was obtained via fluorination of a Schiff-base protected CF_2_Br alanine analog **1**, deprotection of intermediate **2**, and subsequent resolution from its L-[^18^F]-CF_3_-ala isomer via chiral stationary phase HPLC (*%ee* > 90%). (B) *In vitro* bacterial uptake of d-[^18^F]-CF_3_-ala in Gram-positive and Gram-negative pathogens after 90 min incubation. (C) Competition of d-[^18^F]-CF_3_-ala uptake with increasing concentrations of unlabeled (cold) d-ala in *E. coli*.

Based on these promising studies, we developed a radiosynthesis of d-[^18^F]-CF_3_-ala (Figure 4A). The precursor for d-[^18^F]-CF_3_-ala was obtained via alkylation of a glycine-derived Schiff-base with CF_2_Br_2_. The −CF_2_Br precursor (**1**) was then reacted with K_222_/K[^18^F] at 60 °C for 15 min to synthesize intermediate (**2**), which was purified via semipreparative HPLC purification. Further deprotection of (**2**) with TFA at 100 °C provided the racemic [^18^F]-CF_3_-alanine product, which was injected onto a chiral semipreparative HPLC column to isolate d-[^18^F]-CF_3_-ala with 36.3 ± 5.1% RCY (decay corrected), %ee = 90.5 ± 1.7%, RCP > 99%, and *A*_m_ = 0.036 ± 0.004 GBq/μmol (*n* = 11) (Figure 4A). This modest molar activity was attributed to the first step radiofluorination of (**1**), which has been previously observed for CF_2_Br precursors.^29^ This radiosynthesis of d-[^18^F]-CF_3_-ala was used for subsequent *in vitro* and *in vivo* studies.

### d-[^18^F]-CF_3_-ala Was Stable in Serum and Showed Uptake by Numerous Gram-Negative Pathogens *In Vitro*

We tested the stability of d-[^18^F]-CF_3_-ala in mouse serum, human serum and PBS at 37 °C. The d-[^18^F]-CF_3_-ala tracer was stable over 2 h in all solutions (Figure S8), analogous to the results described above for the nonradioactive molecule. We then assessed the uptake of d-[^18^F]-CF_3-_ala into 11 Gram-positive and Gram-negative pathogens *in vitro* (Figure 4B). There was high uptake of the tracer in both *E. coli* and *Acinetobacter baumannii* and significant accumulation in *Enterococcus faecalis*, *Klebsiella pneumoniae*, *P. aeruginosa*, and *Enterobacter cloacae*. We observed lower uptake of the tracer in *S. aureus*, *Listeria monocytogenes*, *Staphylococcus epidermidis*, *Salmonella typhimurium*, and *Proteus mirabilis*. In contrast, no uptake above background signals was observed when d-[^18^F]-CF_3_-ala was incubated with heat-killed *S. aureus* and *E. coli* (Figure S9). The efflux of d-[^18^F]-CF_3-_ala in *E. coli* was evaluated by incubating bacteria with the tracer, followed by washing and incubating in radiotracer-free media. High residual activity (>60%) was seen 30 min after washing the bacteria. (Figure S10).

### PET Using d-[^18^F]-CF_3_-ala Showed Low Background in Normal Animals and Robust Uptake in a Murine Model of Bacterial Infection

**Figure 5:**
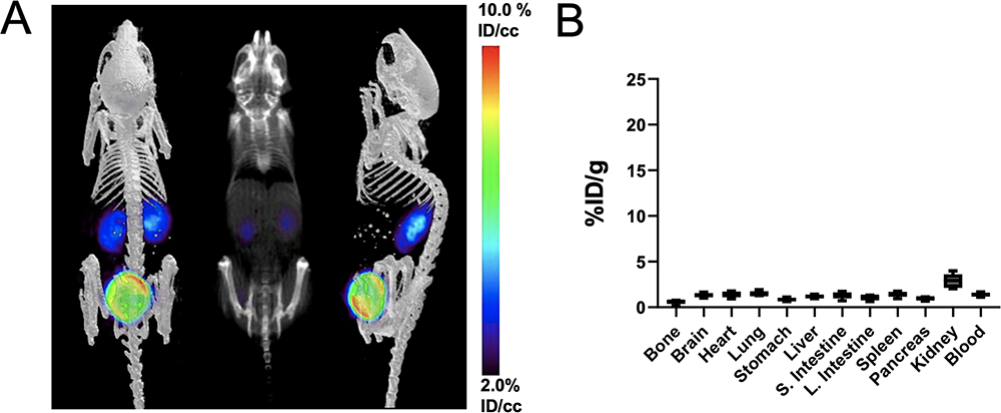
μPET-CT imaging of with d-[^18^F]-CF_3_-ala in mice and *ex vivo* analysis of tracer biodistribution. (A) Maximum intensity projection (MIP) sagittal image of a normal mouse after intravenous administration of d-[^18^F]-CF_3_-ala showing uptake in the kidneys and bladder (*N* = 5). (B) Subsequent *ex vivo* biodistribution analysis using gamma counting of harvested tissues for selected organs.

**Figure 6:**
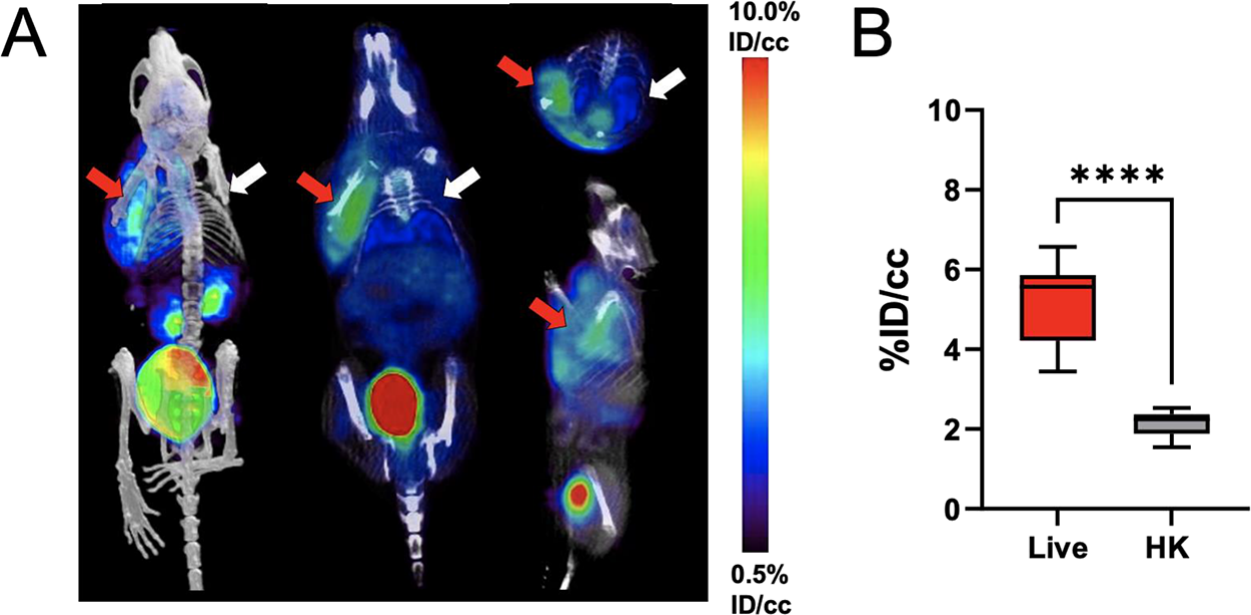
μPET/CT imaging analysis of d-[^18^F]-CF_3_-ala in *E. coli*-infected mice. μPET-CT imaging of *E. coli* myositis in mice with d-[^18^F]-CF_3_-ala (*N* = 8). The red arrows indicate the site of inoculation with live bacteria, while the white arrows correspond to heat-killed bacteria (A). The corresponding bar graphs indicate region-of-interest (ROI) analysis (B). As reflected by the images, the mean d-[^18^F]-CF_3_-ala accumulation for tissues infected with live bacteria was respectively 2.4-fold higher (*P* value < 0.0001) than seen for heat-killed inoculation.

The murine myositis model has proven to be useful for evaluating the characteristics of newly developed PET tracers. In this model, PET tracer accumulation in infection (live bacteria) is compared to that in sterile inflammation (heat-killed bacteria).^2,3,7,15,19^ We first tested the newly developed probe d-[^18^F]-CF_3_-ala in noninfected mice and demonstrated clear renal excretion with low background signal (<2% ID/g in all organs studied with the exception of the kidneys; Figure 5). At 90 min after intravenous injection, the tracer was present only in the kidney and bladder. This result contrasted with the *in vivo* performance of d-[3-^11^C]-ala, which showed uptake in the lungs, pancreas, liver, and kidney in noninfected mice (Figure S11). d-[^18^F]-CF_3_-ala was then evaluated in a murine myositis model in which the mice were inoculated with viable *E. coli* in the left shoulder and heat killed *E. coli* in the right shoulder. d-[^18^F]-CF_3_-ala accumulated at the site of inoculation with live bacteria not heat-killed bacteria, suggesting that this tracer can distinguish infection from sterile inflammation, as corroborated by *ex vivo* analysis. (Figure 6; Figure S12).

## Discussion

A major challenge in detecting bacteria using PET is host tracer metabolism. Although d-[3-^11^C]-alanine is taken up by many microorganisms, its potential conversion to pyruvate by DAAO represents a significant source of background signals *in vivo*. This same consideration applies to other PET tracers used to image infection, including [^11^C]PABA^3^, [^18^F]PABA^6^, and [^18^F]FDS^2^. Mammalian conversion of carbon-11 and fluorine-18 PABA derivatives is a concern given PABA metabolism in the liver to *N*-acetyl PABA, *para*-amino hippuric acid, and *N*-acetyl-*para*-amino hippuric acid by the enzymes *N*-acetyl transferase and glycine *N*-acetyltranferase.^37,38^ Interestingly, although d-sorbitol is metabolized by mammals,^39^ it is fluorine-18 analog 2-deoxy-2-[^18^F]-fluoro-d-sorbitol ([^18^F]FDS) that shows low background signals *in vivo*,^2,40^ suggesting that 2-position fluorination of d-sorbitol arrests mammalian incorporation. We reasoned that the fluorine modification of d-amino acids might be similarly beneficial if this alteration allowed bacterial muropeptide labeling without mammalian oxidation. Despite the similar size of d-[^19^F]-CF_3_-ala versus native d-alanine, the highly electron-withdrawing nature of fluorine would be expected to alter enzyme binding and turnover. To our knowledge, the effects of fluorine substitution on DAAO catalysis have not been previously studied, although fluorinated amino acid side chains may enhance protein binding^41^ and potentially impede product release (the rate-limiting step of DAAO catalysis^42^). NMR data using d-[^19^F]-CF_3_-ala indicated that it is a poor substrate for mammalian conversion via DAAO, and HPLC/MS data showed that exogenous d-[^19^F]-CF_3_-ala could be incorporated into bacterial peptidoglycan. While these studies are not confirmatory of the putative d-[^18^F]-CF_3_-ala bacterial incorporation mechanism due to the differences in metabolite concentrations and labeling times employed, they did suggest that specific labeling of bacteria was possible using the fluorine-18 tracer. Interestingly, imaging using d-[^18^F]-CF_3_-ala showed markedly reduced background signals versus d-[3-^11^C]-alanine and similar uptake by normal organs in comparison to other successful fluorine-18 bacteria-specific tracers.^2,7,9,43^ The infected tissue/organ ratio improved versus d-[3-^11^C]-alanine in several cases, most notably the liver. This lower background of d-[^18^F]-CF_3_-ala would be particularly important in imaging infections of the hepatobiliary system including liver abscesses,^44^ cholangitis,^45^ and acute pancreatitis.^46^ Our studies support variable DAAO oxidation as the source of this difference in background PET signals, although additional experiments should be performed to fully characterize the metabolism of exogenous d-[3-^11^C]-alanine and d-[^18^F]-CF_3_-ala using radio-HPLC^47^ and related methods.

As a significant limitation, the sensitivity of d-[^18^F]-CF_3_-ala for several pathogens in particular Gram-positive bacteria was lower than that seen for d-[3-^11^C]-alanine. We speculate that this low uptake of d-[^18^F]-CF_3_-ala by Gram-positive bacteria may be related to the low molar activity obtained (*A*_m_ = 0.036 ± 0.004 GBq/ μmol) and potentially the lower tolerance of species-specific extra cytoplasmic transpeptidases for the fluorinated substrate. The observed d-[^18^F]-CF_3_-ala molar activity indicated the formation of large quantities of d-[^19^F]-CF_3_-ala from a cold fluoride source, which must have been the CF_2_Br precursor itself providing ^19^F via some disproportionation reaction. In contrast, the molar activity of d-[3-^11^C]-alanine was higher but could not be explicitly calculated due to nondetectable unlabeled d-alanine in the tracer sample.^19^ Despite the advances in [^18^F]trifluoromethylation chemistry previously mentioned,^29^ based on our results using nucleophilic displacement of CF_2_Br precursor **1**, the d-[^18^F]-CF_3_-ala target may not be amenable to late-stage fluorine-18 incorporation. In other words, higher molar activity radiolabeling of an intermediate, followed by subsequent synthetic manipulation, may be required. Another approach is the radiosynthesis and investigation of other fluorinated d-alanine analogs (Figure 1B) that may be synthesized with selective deuterium enrichment to increase their metabolic stability.^48^ These approaches may result in higher sensitivity fluorine-18 radiopharmaceuticals with preserved selectivity for muropeptide functionalization. For future clinical use, the lower uptake of d-[^18^F]-CF_3_-ala in Gram-positive organisms might be beneficial. The relative specificity of d-[^18^F]-CF_3_-ala for Gram-negative bacteria is potentially advantageous for identifying the pathogen type *in vivo* and tailoring appropriate antimicrobial management.

## Conclusions

We have developed a fluorine-18 analogue of d-alanine, d-[^18^F]-CF_3_-ala, that is sensitive to numerous bacterial pathogens including *E. coli* and shows low background in mammalian tissues. The nonradioactive analog d-[^19^F]-CF_3_-ala robustly labels monomeric and dimeric peptides in *E. coli* peptidoglycan and is not a substrate for mammalian d-amino oxidase, suggesting an origin of the observed d-[^18^F]-CF_3_-ala microbial selectivity *in vivo*. Methods to improve the molar activity of d-[^18^F]-CF_3_-ala and incorporate fluorine-18 into other stable d-amino acid scaffolds are anticipated to yield radiopharmaceuticals compatible with human imaging. Furthermore, these results show the power of fluorine-18 modification to selectively observe metabolic processes of interest using PET.

## Materials and Methods

### NMR Analyses

^1^H and ^19^F NMR spectra of d-[^19^F]-CF_3_-ala were obtained on a Bruker Avance III HD 400 MHz instrument at the UCSF Nuclear Magnetic Resonance Laboratory, and data were processed using MestReNova. d-alanine and d-[^19^F]-CF_3_-ala were incubated with DAAO/catalase in 1× PBS with serial ^1^H and ^19^F spectra obtained for 2 h. Stock solutions of 1 mM d-alanine and d--[^19^F]-CF_3_-ala in PBS were prepared and analyzed using ^1^H and ^19^F NMR. A stock solution of enzymes DAAO (4.25 mg/mL) and catalase (0.25 mg/mL) in PBS was prepared and added to NMR tubes containing d-alanine and d-[^19^F]-CF_3_-ala before ^1^H and ^19^F NMR analysis over time. For NMR stability of d-[^19^F]-CF_3_-ala in mouse and human sera, see the Supporting Information for details.

### Peptidoglycan-Labeling

*E. coli* strains BW25113 (wild-type) and BW25113Δ6LDT^49^ were grown in two 100 mL LB media cultures at 37 °C to an OD_600_ of 0.2. To one culture of each strain, 1 mM d-[^18^F]-CF_3_-ala was added, the other culture served as control, and culturing was continued until an OD_600_ of 0.8 was reached. The peptidoglycan was isolated and digested with cellosyl, and the resulting muropeptides were analyzed as previously described.^50^ Briefly, the cultures were cooled on ice for 10 min and cells were retrieved by centrifugation at 4,000 × *g* for 15 min at 4 °C. The cells were resuspended in 6 mL of ice-cold phosphate buffered saline, and the suspension was dropped into 6 mL of boiling 8% SDS under vigorous stirring. Samples were boiled for a further 30 min. The peptidoglycan was pelleted by ultracentrifugation at 400,000 × *g* for 1 h. The pellets were washed free of SDS by sequential resuspension in sterile distilled water and ultracentrifugation until the supernatants were free of SDS, tested as published.^51^ The peptidoglycan was resuspended in 10 mM Tris/HCl pH7.0, incubated with 100 μg/mL amylase for 2 h at 37 °C, followed by incubation for 1 h with 200 μg/mL pronase at 60 °C, and then boiled for 30 min in 4% SDS. Samples were again washed free of SDS and stored at 4 °C with 0.02% sodium azide in water.

The peptidoglycan was digested overnight at 37 °C with 10 μg/mL cellosyl (Hoechst, Germany) in 20 mM sodium phosphate at pH 4.8. The cellosyl was removed by boiling at 100 °C for 10 min and centrifugation at 13,000 × *g* for 10 min. Muropeptides present in the supernatant were reduced with sodium borohydride in 0.25 M sodium borate (pH, 9.0) for 30 min at ambient temperature. The pH was adjusted to 3.5–4.5 using 20% phosphoric acid. Muropeptides were separated on a 250 mm × 4.6 mm Prontosil 3 μm C18 AQ column (Bischoff chromatography) by using an Agilent 1200 series HPLC system. A 140 min linear binary solvent gradient was used using a linear 135 min gradient from 50 mM sodium phosphate pH 4.31 with 0.0001% sodium azide to 75 mM sodium phosphate pH 4.95 with 15% methanol. The column was maintained at 55 °C, and muropeptides were detected by measuring UV absorbance at 205 nm. Fractions corresponding to peaks of interest were collected manually and dried under a vacuum at RT in a Rotovap.

### LC-MS/MS Analysis

For LC-MS/MS analysis, the dried muropeptide sample was reconstituted by addition of 20 μL of 0.2% formic acid (aq) and placed in an autosampler vial. Ten μL of acidified muropeptide was injected onto a microbore RP-HPLC column (ACE 3 C18, 1.0 × 150 mm) flowing at 50 μL min^–1^ delivered by a 1100 HPLC system (Agilent, UK). The column temperature was set at 35 °C, and the first 7 min of eluate was diverted to waste. Buffer A was composed of water containing 0.1% (v/v) formic acid. Buffer B was acetonitrile containing 0.1% (v/v) formic acid. The following elution gradient was used: starting at 0% buffer B, rising to 4% B at 10 min, then on to 5% B at 30 min, rising to 10% B at 53 min, the gradient was ramped to 50% B at 58 min, then on to 85% B at 63 min, followed by a 2 min hold at 85% B, and finally 15 min re-equilibration at 0% B. The total run time was 80 min. The HPLC column eluate was directed to a mass spectrometer (LTQ Ion Trap MS, Thermo) via an IonMax electrospray ion source (Thermo). The settings for the ion source were spray voltage and capillary temperature values of 4,200 V and 200 °C, respectively, together with a sheath gas flow of 1 (arb).

MS data were acquired in positive ion mode over the range of 200–2,000 *m*/*z* in Triple Scan mode. The precursor scan (Enhanced scan rate) was immediately followed by an UltraZoom scan (lower = 3 *m*/*z* units, upper = 5 *m*/*z* units), and finally MS/MS acquisition was performed using a normal scan rate, with activation *Q* = 0.25 and activation time = 30 ms (with wide band activation turned on). The minimum signal threshold was set at 500 counts, MS/MS isolation width set at 2 *m*/*z*, preferred charge state range was set at +1 to +3 and undetermined charge states were excluded. The resulting mass spectral data was opened for analysis using QualBrowser software (Thermo).

### Chemistry and Radiochemistry

Full descriptions of chemical and radiochemical syntheses as well as the analytical techniques used are provided in the Supporting Information. Unless otherwise noted, all of the reagents were obtained commercially and used without further purification. Radioisotopes were generated at the UCSF radiopharmaceutical facility.

### Uptake of d-[^18^F]-CF_3_-ala in Gram-Positive and Gram-Negative Bacteria *In Vitro*

*S. aureus*, *L. monocytogenes*, *S. epidermidis*, *E. faecalis*, *K. pneumoniae*, *E. coli*, *P. aeruginosa*, *A. baumannii*, *S. typhimurium*, *P. mirabilis*, and *E. cloacae* were grown overnight in lysogeny broth (LB) in a shaking incubator at 37 °C. Overnight cultures were diluted to an optical density at 600 nm (OD_600_) of 0.05 and grown to exponential phase (∼0.4–0.6). For uptake studies, bacterial cultures (10 mL) were incubated with 24 μCi of d-[^18^F]-CF_3_-ala at 37 °C for 90 min. After tracer incubation, 500 μL of the bacterial cultures were transferred to Spin-X LC 1.5 mL tubes (0.22 μm) and were centrifuged (6 min, 13200 rpm) to separate bacterial cells and supernatant. Bacterial cells were then washed 1× with phosphate buffered saline (PBS) to remove any tracer not taken up by bacteria. Heat-killed bacterial samples used as a control were prepared by incubating the bacterial cultures at 90 °C for 30 min. Retained radiotracer within samples was then counted using an automated gamma-counter (Hidex). Blocking experiments were performed by adding cold d-ala (0.05 mM to 50 mM) together with 24 μCi of d-[^18^F]-CF_3_-ala following the same protocol. Efflux experiments were performed by incubating the bacteria with 24 μCi of d-[^18^F]-CF_3_-ala for 30 min, then pelleting the bacteria and replacing the media with fresh LB. The cultures were then incubated for an additional 30 min, and then a similar method was used to separate bacteria cells and supernatant. Radioactivity for both was counted using a gamma-counter (HIDEX) to obtain residual activity.

### Animal Experiments

All animal procedures were approved by the UCSF Institutional Animal Care and Use Committee and were performed in accordance with UCSF guidelines. CBA/J mice (female, 8–10 weeks old) were used for the experiments. Mice were housed in individually ventilated cages under normal diet in groups of 5 mice, with ad libitum access to food and water throughout the experiment. Prior to infection and during imaging, the animals were anesthetized with 5% isoflurane. Mice were inoculated in the shoulders with *E. coli* or heat-killed bacteria as described previously,^19^ and imaged using a Inveon μPET-CT following the injection of d-[^18^F]-CF_3_-ala.

### *In vivo* [^18^F]-d-CF_3_-ala Dynamic Imaging in a Myositis Mouse Model

Mice were inoculated with *E. coli* (∼ 2 × 10^7^ colony forming units, CFU) in the left deltoid muscle and 10-fold higher bacterial load of heat-killed bacteria in the right deltoid muscle. After 12h, d-[^18^F]-CF_3_-ala was injected via tail vain (∼ 100 μL, 200 μCi). The mice were then imaged by μPET-CT: whole-body dynamic PET images of healthy or infected mice were obtained for 90 min, followed by a micro-CT scan for 10 min. All data were reconstructed into three-dimensional images to generate dynamic PET images and coregistered with CT images using open-source Amide software.

### Data Analysis and Statistical Considerations

For synthesis, the indicated radiochemical yield incorporates decay-correction for ^18^F (*t*_1/2_ = 109.7 min). *In vitro* data were normalized to CFU’s for sensitivity analysis to account for differential growth rates between organisms. All *in vivo* PET data were viewed by using open-source AMIDE software. Quantification of uptake was performed by drawing spherical regions of interest (5–8 mm^3^) over indicated organs on the CT portion of the exam and expressed as the percent injected dose per gram. All statistical analysis was performed using GraphPad Prism v 9. Data were analyzed using an unpaired two-tailed Student’s *t* test. All graphs are depicted with error bars corresponding to the standard error of the mean.

## Supporting Information Available

Detailed information regarding synthesis, radiosynthesis, *in vitro*, and *in vivo* experiments not reported in the main text (PDF)

## References

[ref1] Polvoy I.; Flavell R. R.; Rosenberg O. S.; Ohliger M. A.; Wilson D. M. Nuclear Imaging of Bacterial Infection: The State of the Art and Future Directions. J. Nucl. Med. 2020, 61 (12), 1708–1716. 10.2967/jnumed.120.244939. 32764120 PMC9364899

[ref2] Weinstein E. A.; Ordonez A. A.; DeMarco V. P.; Murawski A. M.; Pokkali S.; MacDonald E. M.; Klunk M.; Mease R. C.; Pomper M. G.; Jain S. K. Imaging Enterobacteriaceae Infection in Vivo with 18F-Fluorodeoxysorbitol Positron Emission Tomography. Sci. Transl. Med. 2014, 6 (259), 259ra146 10.1126/scitranslmed.3009815. PMC4327834 25338757

[ref3] Mutch C. A.; Ordonez A. A.; Qin H.; Parker M.; Bambarger L. E.; Villanueva-Meyer J. E.; Blecha J.; Carroll V.; Taglang C.; Flavell R.; Sriram R.; VanBrocklin H.; Rosenberg O.; Ohliger M. A.; Jain S. K.; Neumann K. D.; Wilson D. M. [11C]Para-Aminobenzoic Acid: A Positron Emission Tomography Tracer Targeting Bacteria-Specific Metabolism. ACS Infect. Dis. 2018, 4 (7), 1067–1072. 10.1021/acsinfecdis.8b00061. 29712422 PMC6045447

[ref4] Gowrishankar G.; Hardy J.; Wardak M.; Namavari M.; Reeves R. E.; Neofytou E.; Srinivasan A.; Wu J. C.; Contag C. H.; Gambhir S. S. Specific Imaging of Bacterial Infection Using 6″-18F-Fluoromaltotriose: A Second-Generation PET Tracer Targeting the Maltodextrin Transporter in Bacteria. J. Nucl. Med. 2017, 58 (10), 1679–1684. 10.2967/jnumed.117.191452. 28490473 PMC5632736

[ref5] Sellmyer M. A.; Lee I.; Hou C.; Weng C.-C.; Li S.; Lieberman B. P.; Zeng C.; Mankoff D. A.; Mach R. H. Bacterial Infection Imaging with [18F]Fluoropropyl-Trimethoprim. Proc. Natl. Acad. Sci. U. S. A. 2017, 114 (31), 8372–8377. 10.1073/pnas.1703109114. 28716936 PMC5547613

[ref6] Zhang Z.; Ordonez A. A.; Wang H.; Li Y.; Gogarty K. R.; Weinstein E. A.; Daryaee F.; Merino J.; Yoon G. E.; Kalinda A. S.; Mease R. C.; Iuliano J. N.; Smith-Jones P. M.; Jain S. K.; Tonge P. J. Positron Emission Tomography Imaging with 2-[18F]F- p-Aminobenzoic Acid Detects Staphylococcus Aureus Infections and Monitors Drug Response. ACS Infect. Dis. 2018, 4 (11), 1635–1644. 10.1021/acsinfecdis.8b00182. 30067329 PMC6226330

[ref7] Sorlin A. M.; López-Álvarez M.; Rabbitt S. J.; Alanizi A. A.; Shuere R.; Bobba K. N.; Blecha J.; Sakhamuri S.; Evans M. J.; Bayles K. W.; Flavell R. R.; Rosenberg O. S.; Sriram R.; Desmet T.; Nidetzky B.; Engel J.; Ohliger M. A.; Fraser J. S.; Wilson D. M. Chemoenzymatic Syntheses of Fluorine-18-Labeled Disaccharides from [18F] FDG Yield Potent Sensors of Living Bacteria In Vivo. J. Am. Chem. Soc. 2023, 145 (32), 17632–17642. 10.1021/jacs.3c03338. 37535945 PMC10436271

[ref8] Ning X.; Seo W.; Lee S.; Takemiya K.; Rafi M.; Feng X.; Weiss D.; Wang X.; Williams L.; Camp V. M.; Eugene M.; Taylor W. R.; Goodman M.; Murthy N. PET Imaging of Bacterial Infections with Fluorine-18-Labeled Maltohexaose. Angew. Chem. Int. Ed 2014, 53 (51), 14096–14101. 10.1002/anie.201408533. PMC4430476 25330976

[ref9] Simpson S. R.; Kesterson A. E.; Wilde J. H.; Qureshi Z.; Kundu B.; Simons M. P.; Neumann K. D. Imaging Diverse Pathogenic Bacteria In Vivo with 18F-Fluoromannitol PET. J. Nucl. Med. 2023, 64 (5), 809–815. 10.2967/jnumed.122.264854. 36522188 PMC10152124

[ref10] Petrik M.; Haas H.; Schrettl M.; Helbok A.; Blatzer M.; Decristoforo C. In Vitro and in Vivo Evaluation of Selected 68Ga-Siderophores for Infection Imaging. Nucl. Med. Biol. 2012, 39 (3), 361–369. 10.1016/j.nucmedbio.2011.09.012. 22172389 PMC3314960

[ref11] Petrik M.; Umlaufova E.; Raclavsky V.; Palyzova A.; Havlicek V.; Haas H.; Novy Z.; Dolezal D.; Hajduch M.; Decristoforo C. Imaging of Pseudomonas Aeruginosa Infection with Ga-68 Labelled Pyoverdine for Positron Emission Tomography. Sci. Rep. 2018, 8 (1), 15698 10.1038/s41598-018-33895-w. 30356077 PMC6200719

[ref12] Kuru E.; Tekkam S.; Hall E.; Brun Y. V.; Van Nieuwenhze M. S. Synthesis of Fluorescent D-Amino Acids and Their Use for Probing Peptidoglycan Synthesis and Bacterial Growth in Situ. Nat. Protoc. 2015, 10 (1), 33–52. 10.1038/nprot.2014.197. 25474031 PMC4300143

[ref13] Hsu Y.-P.; Rittichier J.; Kuru E.; Yablonowski J.; Pasciak E.; Tekkam S.; Hall E.; Murphy B.; Lee T. K.; Garner E. C.; Huang K. C.; Brun Y. V.; VanNieuwenhze M. S. Full Color Palette of Fluorescent D-Amino Acids for in Situ Labeling of Bacterial Cell Walls. Chem. Sci. 2017, 8 (9), 6313–6321. 10.1039/C7SC01800B. 28989665 PMC5628581

[ref14] Siegrist M. S.; Whiteside S.; Jewett J. C.; Aditham A.; Cava F.; Bertozzi C. R. (D)-Amino Acid Chemical Reporters Reveal Peptidoglycan Dynamics of an Intracellular Pathogen. ACS Chem. Biol. 2013, 8 (3), 500–505. 10.1021/cb3004995. 23240806 PMC3601600

[ref15] Neumann K. D.; Villanueva-Meyer J. E.; Mutch C. A.; Flavell R. R.; Blecha J. E.; Kwak T.; Sriram R.; VanBrocklin H. F.; Rosenberg O. S.; Ohliger M. A.; Wilson D. M. Imaging Active Infection in Vivo Using D-Amino Acid Derived PET Radiotracers. Sci. Rep. 2017, 7 (1), 7903 10.1038/s41598-017-08415-x. 28801560 PMC5554133

[ref16] Polvoy I.; Seo Y.; Parker M.; Stewart M.; Siddiqua K.; Manacsa H. S.; Ravanfar V.; Blecha J.; Hope T. A.; Vanbrocklin H.; Flavell R. R.; Barry J.; Hansen E.; Villanueva-Meyer J. E.; Engel J.; Rosenberg O. S.; Wilson D. M.; Ohliger M. A. Imaging Joint Infections Using D-Methyl-11C-Methionine PET/MRI: Initial Experience in Humans. Eur. J. Nucl. Med. Mol. Imaging 2022, 49 (11), 3761–3771. 10.1007/s00259-022-05858-x. 35732972 PMC9399217

[ref17] Stewart M. N.; Parker M. F. L.; Jivan S.; Luu J. M.; Huynh T. L.; Schulte B.; Seo Y.; Blecha J. E.; Villanueva-Meyer J. E.; Flavell R. R.; VanBrocklin H. F.; Ohliger M. A.; Rosenberg O.; Wilson D. M. High Enantiomeric Excess In-Loop Synthesis of d-[Methyl-11C]Methionine for Use as a Diagnostic Positron Emission Tomography Radiotracer in Bacterial Infection. ACS Infect. Dis. 2020, 6 (1), 43–49. 10.1021/acsinfecdis.9b00196. 31697062 PMC7364312

[ref18] Renick P. J.; Mulgaonkar A.; Co C. M.; Wu C.-Y.; Zhou N.; Velazquez A.; Pennington J.; Sherwood A.; Dong H.; Castellino L.; Öz O. K.; Tang L.; Sun X. Imaging of Actively Proliferating Bacterial Infections by Targeting the Bacterial Metabolic Footprint with D-[5–11C]-Glutamine. ACS Infect. Dis. 2021, 7 (2), 347–361. 10.1021/acsinfecdis.0c00617. 33476123

[ref19] Parker M. F. L.; Luu J. M.; Schulte B.; Huynh T. L.; Stewart M. N.; Sriram R.; Yu M. A.; Jivan S.; Turnbaugh P. J.; Flavell R. R.; Rosenberg O. S.; Ohliger M. A.; Wilson D. M. Sensing Living Bacteria in Vivo Using D-Alanine-Derived 11C Radiotracers. ACS Cent. Sci. 2020, 6 (2), 155–165. 10.1021/acscentsci.9b00743. 32123733 PMC7047270

[ref20] Parker M. F. L.; López-Álvarez M.; Alanizi A. A.; Luu J. M.; Polvoy I.; Sorlin A. M.; Qin H.; Lee S.; Rabbitt S. J.; Pichardo-González P. A.; Ordonez A. A.; Blecha J.; Rosenberg O. S.; Flavell R. R.; Engel J.; Jain S. K.; Ohliger M. A.; Wilson D. M. Evaluating the Performance of Pathogen-Targeted Positron Emission Tomography Radiotracers in a Rat Model of Vertebral Discitis-Osteomyelitis. J. Infect. Dis. 2023, 228 (Suppl 4), S281–S290. 10.1093/infdis/jiad159. 37788505 PMC11009497

[ref21] Fura J. M.; Kearns D.; Pires M. M. D-Amino Acid Probes for Penicillin Binding Protein-Based Bacterial Surface Labeling. J. Biol. Chem. 2015, 290 (51), 30540–30550. 10.1074/jbc.M115.683342. 26499795 PMC4683274

[ref22] Wang L.; Zha Z.; Qu W.; Qiao H.; Lieberman B. P.; Plössl K.; Kung H. F. Synthesis and Evaluation of 18F Labeled Alanine Derivatives as Potential Tumor Imaging Agents. Nucl. Med. Biol. 2012, 39 (7), 933–943. 10.1016/j.nucmedbio.2012.03.007. 22542392 PMC3432733

[ref23] Yang D.; Kuang L. R.; Cherif A.; Tansey W.; Li C.; Lin W. J.; Liu C. W.; Kim E. E.; Wallace S. Synthesis of [18F]Fluoroalanine and [18F]Fluorotamoxifen for Imaging Breast Tumors. J. Drug Target. 1993, 1 (3), 259–267. 10.3109/10611869308996084. 8069568

[ref24] Pollegioni L.; Sacchi S.; Murtas G. Human D-Amino Acid Oxidase: Structure, Function, and Regulation. Front. Mol. Biosci. 2018, 5, 107 10.3389/fmolb.2018.00107. 30547037 PMC6279847

[ref25] Radaelli A.; Gruetter R.; Yoshihara H. A. I. In Vivo Detection of D-Amino Acid Oxidase with Hyperpolarized d-[1–13 C]Alanine. NMR Biomed. 2020, 33 (7), e4303 10.1002/nbm.4303. 32325540

[ref26] Kuru E.; Radkov A.; Meng X.; Egan A.; Alvarez L.; Dowson A.; Booher G.; Breukink E.; Roper D. I.; Cava F.; Vollmer W.; Brun Y.; VanNieuwenhze M. S. Mechanisms of Incorporation for D-Amino Acid Probes That Target Peptidoglycan Biosynthesis. ACS Chem. Biol. 2019, 14 (12), 2745–2756. 10.1021/acschembio.9b00664. 31743648 PMC6929685

[ref27] Abula A.; Xu Z.; Zhu Z.; Peng C.; Chen Z.; Zhu W.; Aisa H. A. Substitution Effect of the Trifluoromethyl Group on the Bioactivity in Medicinal Chemistry: Statistical Analysis and Energy Calculations. J. Chem. Inf. Model. 2020, 60 (12), 6242–6250. 10.1021/acs.jcim.0c00898. 33258377

[ref28] Schiesser S.; Chepliaka H.; Kollback J.; Quennesson T.; Czechtizky W.; Cox R. J. N-Trifluoromethyl Amines and Azoles: An Underexplored Functional Group in the Medicinal Chemist’s Toolbox. J. Med. Chem. 2020, 63 (21), 13076–13089. 10.1021/acs.jmedchem.0c01457. 33112606

[ref29] Francis F.; Wuest F. Advances in [18f]Trifluoromethylation Chemistry for PET Imaging. Molecules 2021, 26 (21), 6478 10.3390/molecules26216478. 34770885 PMC8587676

[ref30] Levin M. D.; Chen T. Q.; Neubig M. E.; Hong C. M.; Theulier C. A.; Kobylianskii I. J.; Janabi M.; O’Neil J. P.; Toste F. D. A Catalytic Fluoride-Rebound Mechanism for C(Sp3)-CF3 Bond Formation. Science 2017, 356 (6344), 1272–1276. 10.1126/science.aan1411. 28642435 PMC5902185

[ref31] Huiban M.; Tredwell M.; Mizuta S.; Wan Z.; Zhang X.; Collier T. L.; Gouverneur V.; Passchier J. A Broadly Applicable [18F]Trifluoromethylation of Aryl and Heteroaryl Iodides for PET Imaging. Nat. Chem. 2013, 5 (11), 941–944. 10.1038/nchem.1756. 24153372

[ref32] Fu Z.; Lin Q.; Hu B.; Zhang Y.; Chen W.; Zhu J.; Zhao Y.; Choi H. S.; Shi H.; Cheng D. P2 × 7 PET Radioligand 18F-PTTP for Differentiation of Lung Tumor from Inflammation. J. Nucl. Med. 2019, 60 (7), 930–936. 10.2967/jnumed.118.222547. 30655332 PMC6604685

[ref33] Dolbier W. R.; Li A. R.; Koch C. J.; Shiue C. Y.; Kachur A. V. [18F]-EF5, a Marker for PET Detection of Hypoxia: Synthesis of Precursor and a New Fluorination Procedure. Appl. Radiat. Isot. 2001, 54 (1), 73–80. 10.1016/S0969-8043(00)00102-0. 11144255

[ref34] Szpera R.; Isenegger P. G.; Ghosez M.; Straathof N. J. W.; Cookson R.; Blakemore D. C.; Richardson P.; Gouverneur V. Synthesis of Fluorinated Alkyl Aryl Ethers by Palladium-Catalyzed C-O Cross-Coupling. Org. Lett. 2020, 22 (16), 6573–6577. 10.1021/acs.orglett.0c02347. 32806200 PMC7458480

[ref35] Fawaz M. V.; Brooks A. F.; Rodnick M. E.; Carpenter G. M.; Shao X.; Desmond T. J.; Sherman P.; Quesada C. A.; Hockley B. G.; Kilbourn M. R.; Albin R. L.; Frey K. A.; Scott P. J. H. High Affinity Radiopharmaceuticals Based upon Lansoprazole for PET Imaging of Aggregated Tau in Alzheimer’s Disease and Progressive Supranuclear Palsy: Synthesis, Preclinical Evaluation, and Lead Selection. ACS Chem. Neurosci. 2014, 5 (8), 718–730. 10.1021/cn500103u. 24896980 PMC4140593

[ref36] Turkman N.; Liu D.; Pirola I. Novel Late-Stage Radiosynthesis of 5-[18F]-Trifluoromethyl-1,2,4-Oxadiazole (TFMO) Containing Molecules for PET Imaging. Sci. Rep. 2021, 11 (1), 10668 10.1038/s41598-021-90069-x. 34021207 PMC8139947

[ref37] Laurieri N.; Dairou J.; Egleton J. E.; Stanley L. A.; Russell A. J.; Dupret J.-M.; Sim E.; Rodrigues-Lima F. From Arylamine N-Acetyltransferase to Folate-Dependent Acetyl CoA Hydrolase: Impact of Folic Acid on the Activity of (HUMAN)NAT1 and Its Homologue (MOUSE)NAT2. PLoS One 2014, 9 (5), e96370 10.1371/journal.pone.0096370. 24823794 PMC4019507

[ref38] Furuya K. N.; Durie P. R.; Roberts E. A.; Soldin S. J.; Verjee Z.; Yung-Jato L.; Giesbrecht E.; Ellis L. Glycine Conjugation of Para-Aminobenzoic Acid (PABA): A Quantitative Test of Liver Function. Clin. Biochem. 1995, 28 (5), 531–540. 10.1016/0009-9120(95)00040-G. 8582053

[ref39] Adcock L. H.; Gray C. H. The Metabolism of Sorbitol in the Human Subject. Biochem. J. 1957, 65 (3), 554–560. 10.1042/bj0650554. 13412662 PMC1199912

[ref40] Ordonez A. A.; Wintaco L. M.; Mota F.; Restrepo A. F.; Ruiz-Bedoya C. A.; Reyes C. F.; Uribe L. G.; Abhishek S.; D’Alessio F. R.; Holt D. P.; Dannals R. F.; Rowe S. P.; Castillo V. R.; Pomper M. G.; Granados U.; Jain S. K. Imaging Enterobacterales Infections in Patients Using Pathogen-Specific Positron Emission Tomography. Sci. Transl. Med. 2021, 13 (589), eabe9805 10.1126/scitranslmed.abe9805. 33853931 PMC8120649

[ref41] Miles S. A.; Nillama J. A.; Hunter L. Tinker, Tailor, Soldier, Spy: The Diverse Roles That Fluorine Can Play within Amino Acid Side Chains. Molecules 2023, 28 (17), 6192 10.3390/molecules28176192. 37687021 PMC10489206

[ref42] Molla G.; Sacchi S.; Bernasconi M.; Pilone M. S.; Fukui K.; Polegioni L. Characterization of Human D-Amino Acid Oxidase. FEBS Lett. 2006, 580 (9), 2358–2364. 10.1016/j.febslet.2006.03.045. 16616139

[ref43] Lee S. H.; Kim J. M.; López-Álvarez M.; Wang C.; Sorlin A. M.; Bobba K. N.; Pichardo-González P. A.; Blecha J.; Seo Y.; Flavell R. R.; Engel J.; Ohliger M. A.; Wilson D. M. Imaging the Bacterial Cell Wall Using N-Acetyl Muramic Acid-Derived Positron Emission Tomography Radiotracers. ACS Sens. 2023, 8 (12), 4554–4565. 10.1021/acssensors.3c01477. 37992233 PMC10749472

[ref44] Lardière-Deguelte S.; Ragot E.; Amroun K.; Piardi T.; Dokmak S.; Bruno O.; Appere F.; Sibert A.; Hoeffel C.; Sommacale D.; Kianmanesh R. Hepatic Abscess: Diagnosis and Management. J. Visc. Surg. 2015, 152 (4), 231–243. 10.1016/j.jviscsurg.2015.01.013. 25770745

[ref45] Ahmed M. Acute Cholangitis - an Update. World J. Gastrointest. Pathophysiol. 2018, 9 (1), 1–7. 10.4291/wjgp.v9.i1.1. 29487761 PMC5823698

[ref46] Mederos M. A.; Reber H. A.; Girgis M. D. Acute Pancreatitis: A Review. JAMA 2021, 325 (4), 382–390. 10.1001/jama.2020.20317. 33496779

[ref47] Rokka J.; Grönroos T. J.; Viljanen T.; Solin O.; Haaparanta-Solin M. HPLC and TLC Methods for Analysis of [18F]FDG and Its Metabolites from Biological Samples. J. Chromatogr. B Analyt. Technol. Biomed. Life Sci. 2017, 1048, 140–149. 10.1016/j.jchromb.2017.01.042. 28236580

[ref48] Kuchar M.; Mamat C. Methods to Increase the Metabolic Stability of (18)F-Radiotracers. Molecules 2015, 20 (9), 16186–16220. 10.3390/molecules200916186. 26404227 PMC6332123

[ref49] Kuru E.; Lambert C.; Rittichier J.; Till R.; Ducret A.; Derouaux A.; Gray J.; Biboy J.; Vollmer W.; VanNieuwenhze M.; Brun Y. V.; Sockett R. E. Fluorescent D-Amino-Acids Reveal Bi-Cellular Cell Wall Modifications Important for Bdellovibrio Bacteriovorus Predation. Nat. Microbiol. 2017, 2 (12), 1648–1657. 10.1038/s41564-017-0029-y. 28974693 PMC5705579

[ref50] Glauner B. Separation and Quantification of Muropeptides with High-Performance Liquid Chromatography. Anal. Biochem. 1988, 172 (2), 451–464. 10.1016/0003-2697(88)90468-X. 3056100

[ref51] Hayashi K. A Rapid Determination of Sodium Dodecyl Sulfate with Methylene Blue. Anal. Biochem. 1975, 67 (2), 503–506. 10.1016/0003-2697(75)90324-3. 1163770

