# Supplementary material for: Peptidoglycan-Targeted [18F]3,3,3-Trifluoro-d-alanine Tracer for Imaging Bacterial Infection
Source: JACS Au. 2024 Feb 26;4(3):1039–47. doi: 10.1021/jacsau.3c00776 (PMC10976610; doi:10.1021/jacsau.3c00776)
Supplement: Supplementary file 1 — au3c00776_si_001.pdf [file au3c00776_si_001.pdf]

Supporting Information for:

Peptidoglycan-targeted [^{18}F]3,3,3-trifluoro-D-alanine tracer for imaging bacterial infection

Alexandre M. Sorlin[1], Marina López-Álvarez[1], Jacob Biboy[2], Joe Gray[2], Sarah J. Rabbitt[1], Junaid Ur Rahim[1], Sang Hee Lee[1], Kondapa Naidu Bobba[1], Joseph Blecha[1], Mathew F.L. Parker[1,3], Robert R. Flavell[1,4,5], Joanne Engel[1,6], Michael Ohliger[1,7], Waldemar Vollmer[2,8], David M. Wilson[1*]

[1]Department of Radiology and Biomedical Imaging
University of California, San Francisco
San Francisco, CA 94158, USA

[2]The Centre for Bacterial Cell Biology
Newcastle University
Newcastle, United Kingdom NE2 4AX

[3]Department of Psychiatry
Renaissance School of Medicine at Stony Brook University
Stony Brook, NY, 11794 USA

[4]UCSF Helen Diller Family Comprehensive Cancer Center
University of California, San Francisco
San Francisco, CA 94158, USA

[5]Department of Pharmaceutical Chemistry
University of California, San Francisco
San Francisco, CA 94158, USA

[6]Department of Medicine
University of California, San Francisco
San Francisco, CA 94158, USA

[7]Department of Radiology
Zuckerberg San Francisco General Hospital
San Francisco CA 94110, USA

[8]Institute for Molecular Bioscience
The University of Queensland
Brisbane, 4072, Australia

Table of Contents

A. **Supplemental Figures……………………………………………………………….……2**
B. **Synthetic Procedures………………………………………………………………13**
C. **Radiochemistry…………………………………………………………………………20**
D. **References………………………………………..…………………………………..…23**

A. Supplemental Figures

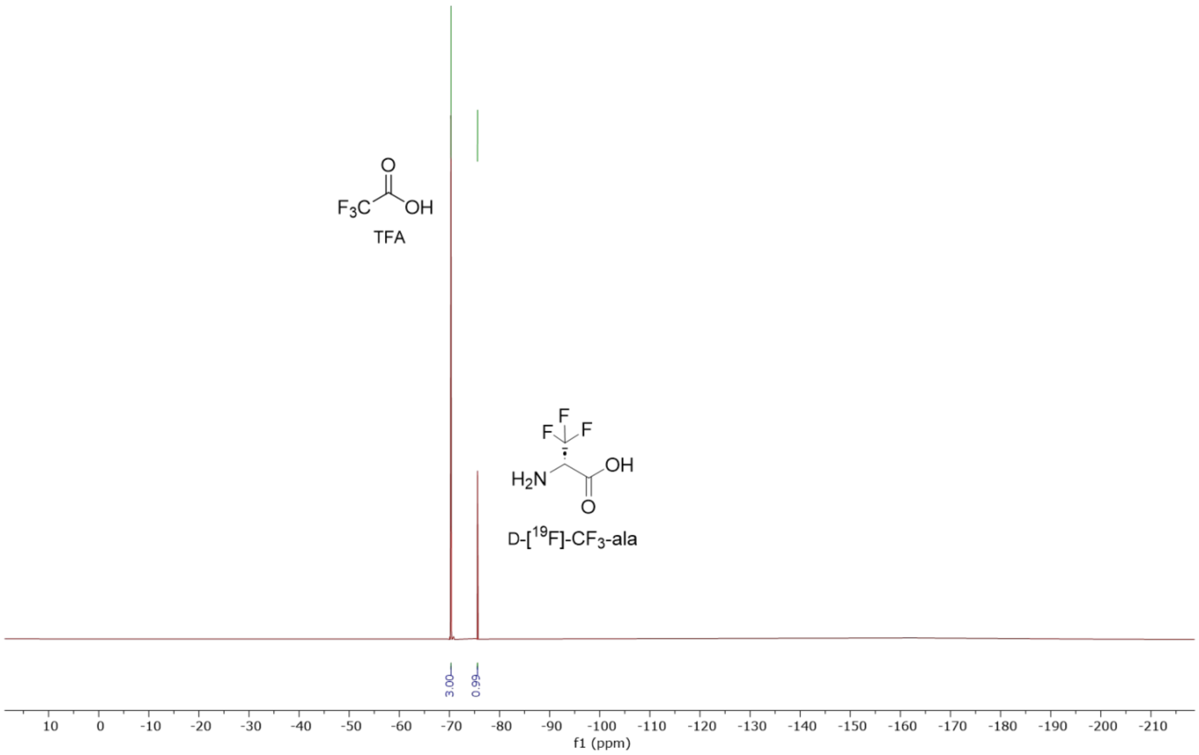

Figure S1. Stability of D-[^{19}F]-CF_3-ala in mouse serum using ^{19}F NMR. In mouse and human sera, D-[^{19}F]-CF_3-ala (0.1M) was stirred for 6 hours at 37°C. At the end of mixing, TFA (3 eq) was added as an internal standard before an aliquot was taken for ^{19}F NMR analysis, which showed no degradation of D-[^{19}F]-CF_3-ala. An identical ^{19}F spectrum was observed for the analogous experiment using human serum.

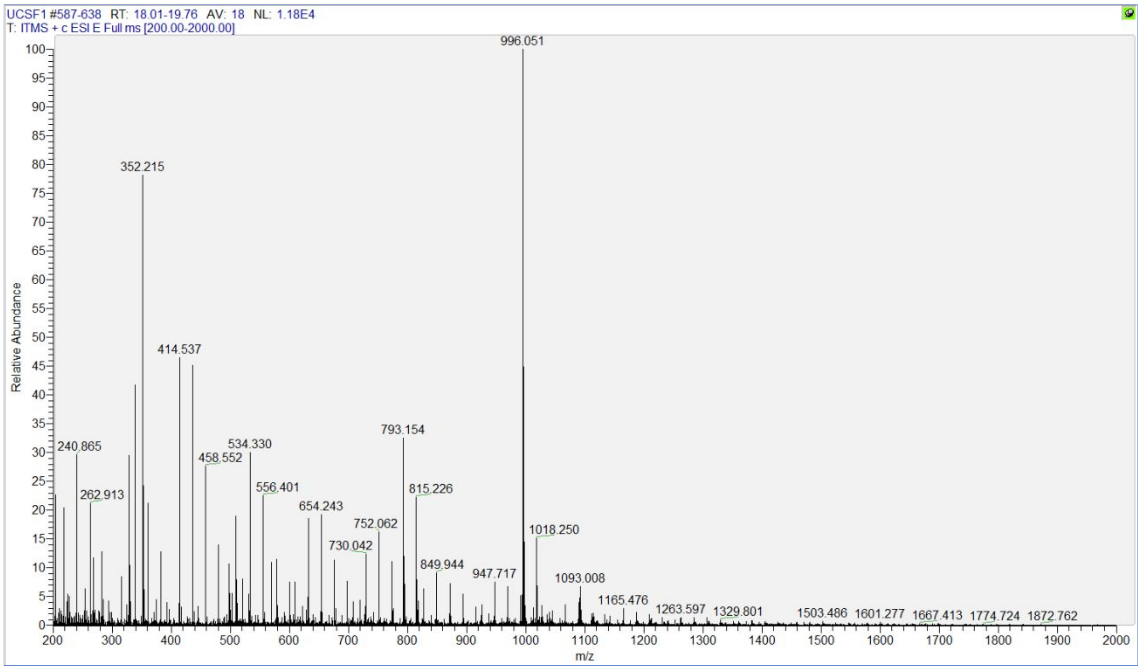

Figure S2. Peak 1 LC-MS – Major ion observed at m/z = 996.05 ($M+H^+$).

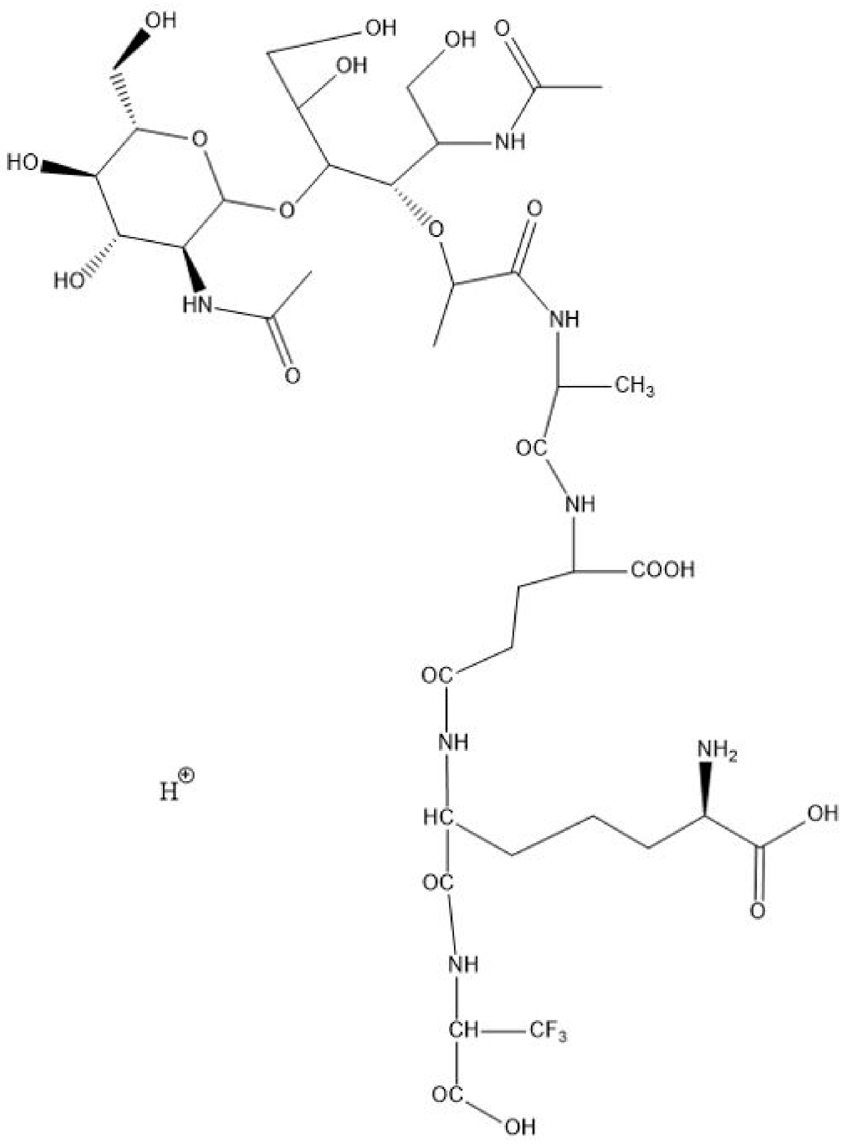

Chemical Formula: $C_{37}H_{61}F_3N_7O_{21}^+$
Exact Mass: 996.39

Figure S3. Peak 1 Predicted Structure – Singly charged ion at predicted m/z = 996.39.

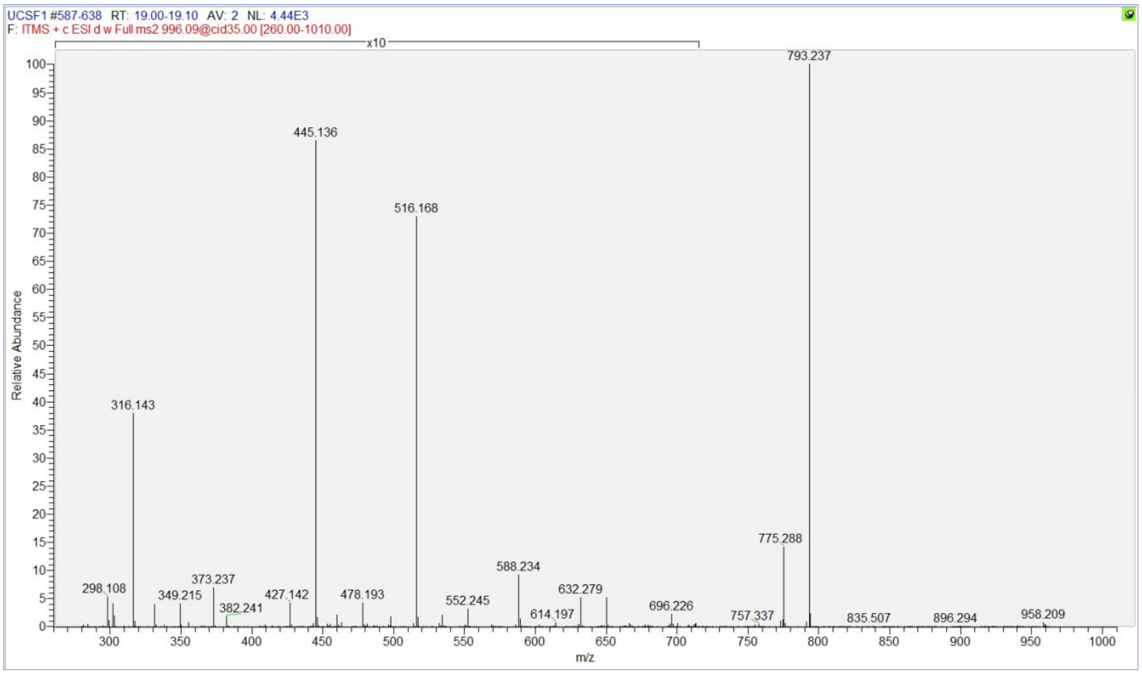

Figure S4. Peak 1 LC-MS/MS (fragment ions).

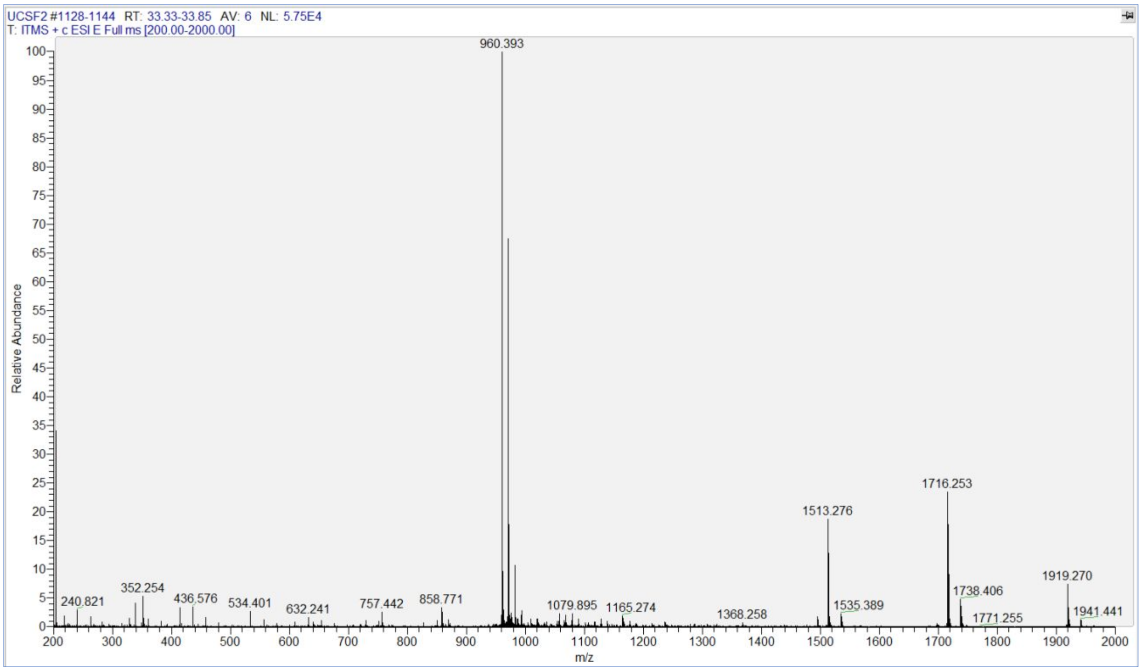

Figure S5. Peak 2 LC-MS - Major ion observed at m/z = 960.39 (M+2H^{++}).

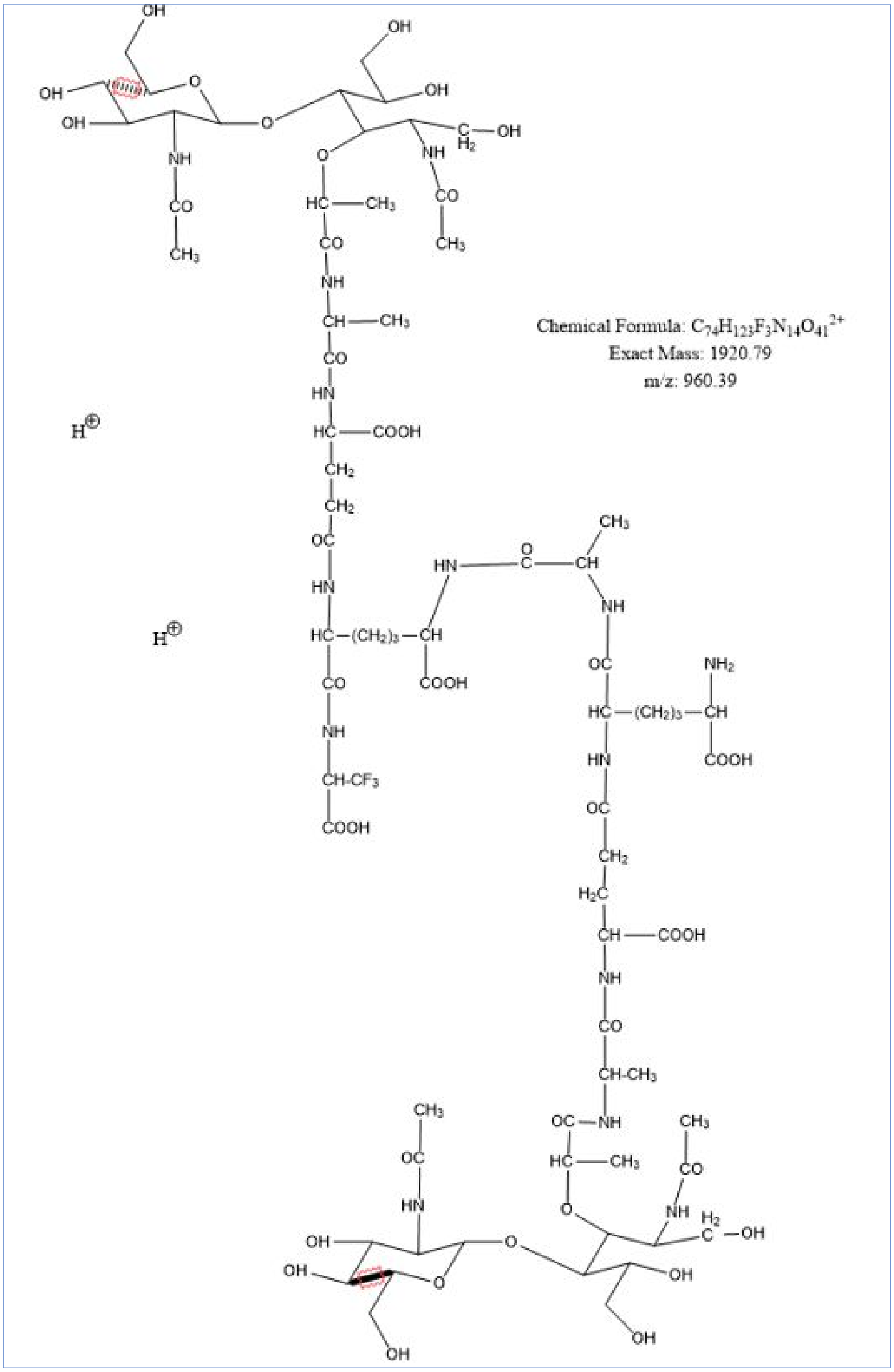

Figure S6. Peak 2 Predicted Structure – Doubly charged ion at predicted m/z = 960.39.

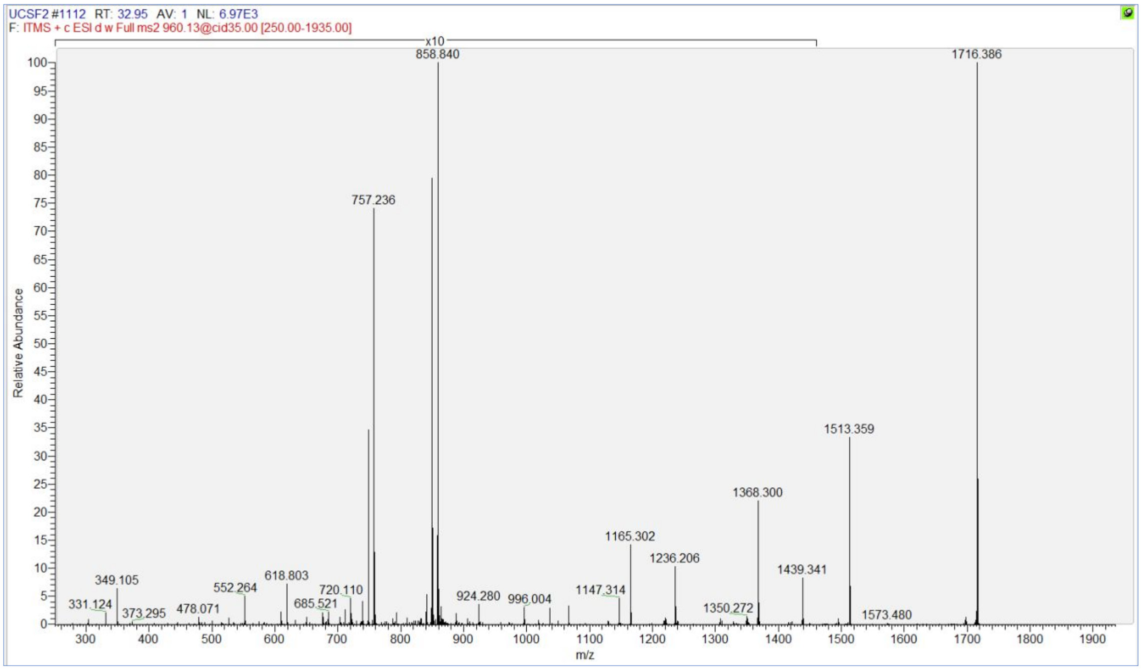

Figure S7. Peak 2 LC-MS/MS (fragment ions).

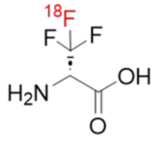

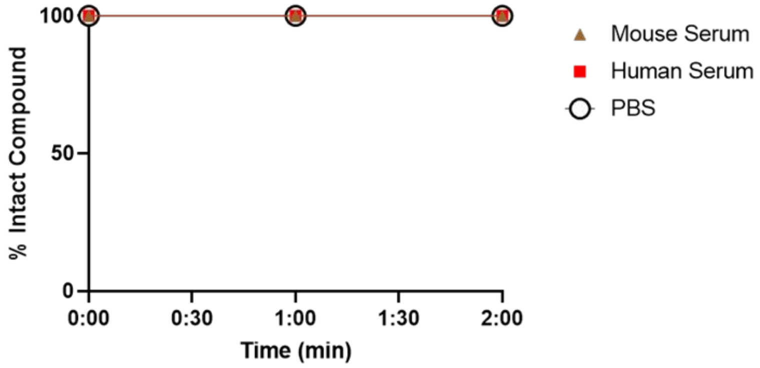

Figure S8. Stability of D-[^{18}F]-CF_3-ala in PBS, human and mouse serum at 37°C over time using Radio TLC, Silica gel, 100% EtOAc.

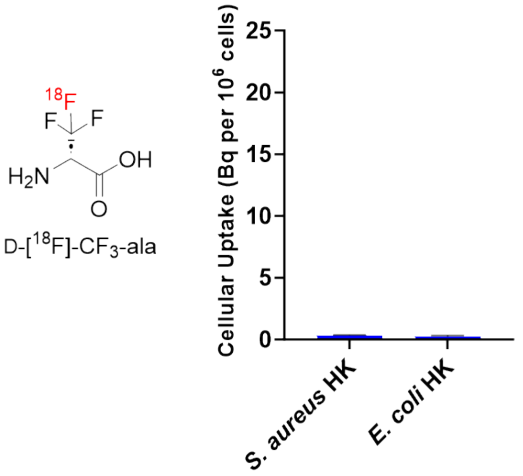

Figure S9. *In vitro* uptake of D-[^{18}F]-CF_3-ala in heat-killed bacteria.

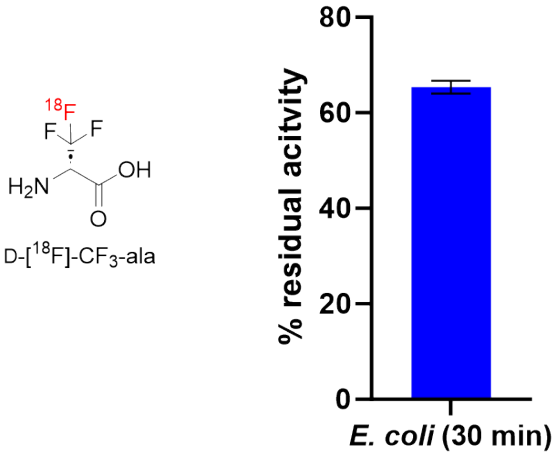

Figure S10. Efflux experiment. An *E. coli* culture was initially incubated with D-[^{18}F]-CF_3-ala for 30 minutes @ 37 °C in LB and washed in 1X PBS (x3). The pellet was isolated using Spin-X LC 1.5 mL tubes with the retained activity measured. This washing and counting procedure took about 10 minutes. The bacteria were subsequently re-suspended in LB without tracer for an

additional 30 minutes, and washed/counted as above. Plot shows the residual (decay-adjusted) activity.

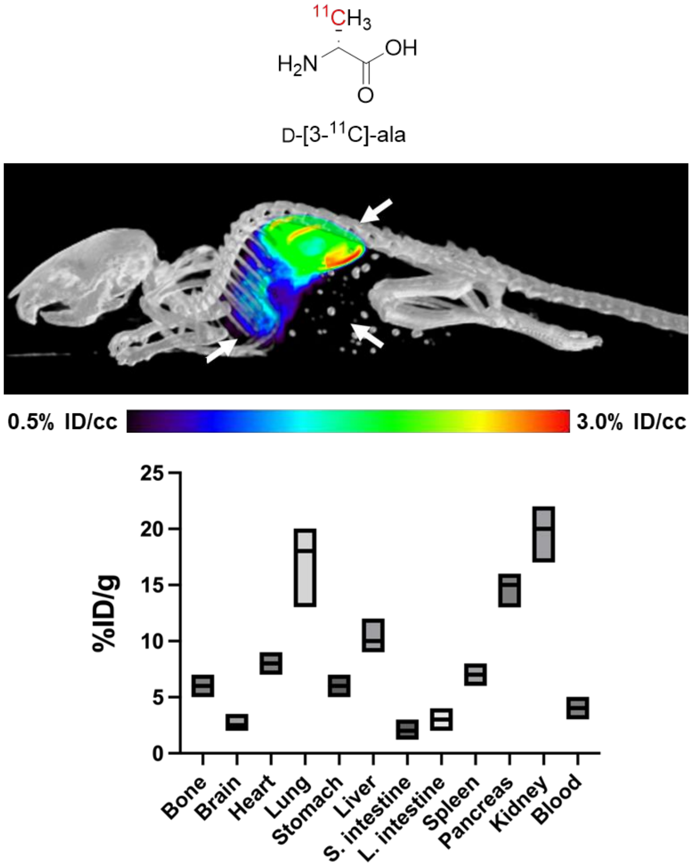

Figure S11. *In vivo* experiment: D-[^{11}C]-ala in mouse, no bacteria. Reproduced with permission from Parker et al.[1] Copyright American Chemical Society.

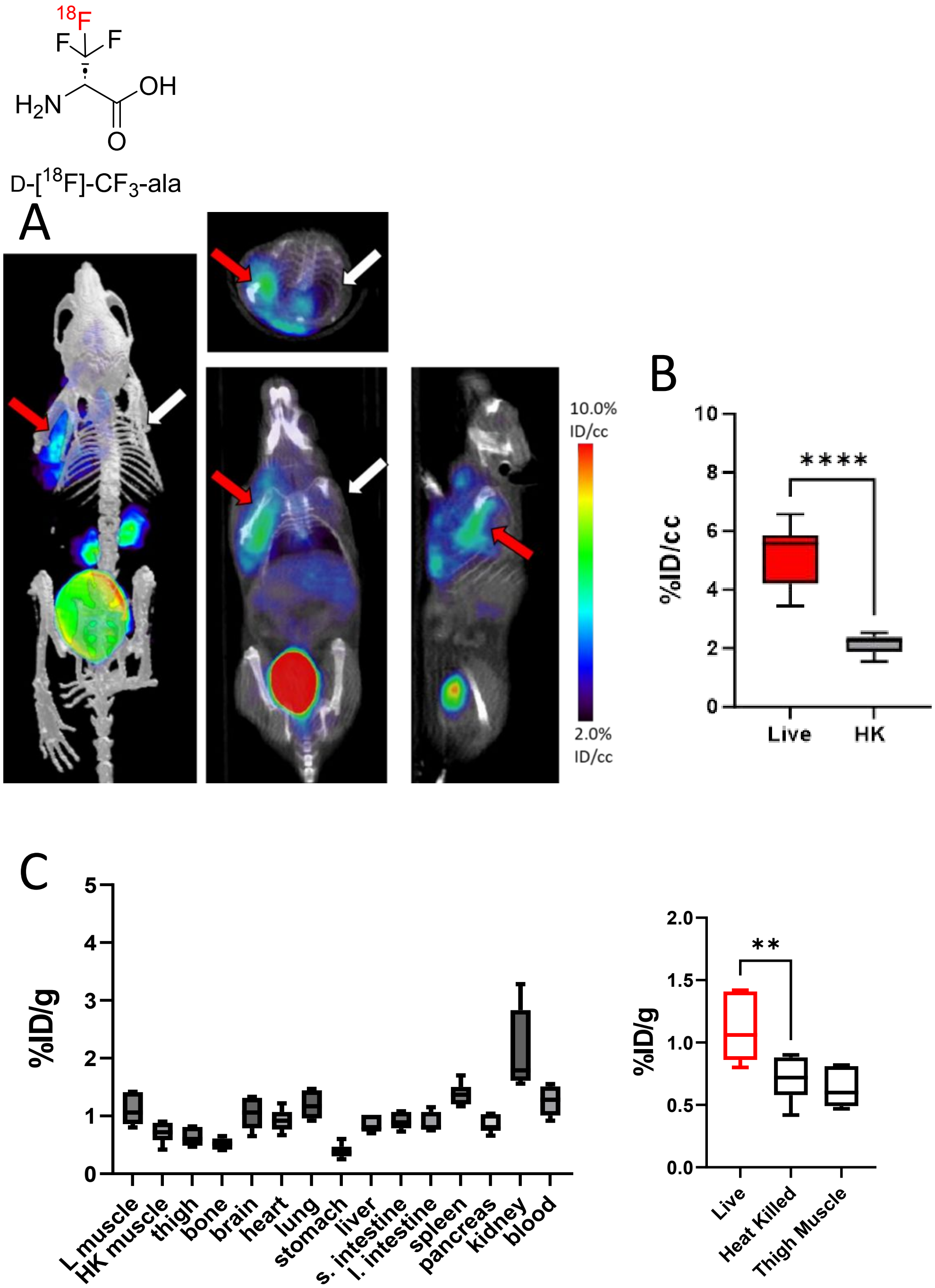

Figure S12. *In vivo* experiment: D-[^{18}F]-CF_3-ala in mouse myositis model, *E. coli* (ATCC: 25922), Injection: ≈200uCi ^{18}F-tracer, N = 8. (A) The red arrows indicate the site of inoculation with live bacteria, while white arrows indicate the site of inoculation with heat-killed bacteria. (B) ROI analysis, *live vs HK*: 2.4-fold excess, **** *P* value <0.0001, (C) *Ex vivo* data was obtained following tissue harvesting on a gamma counter, *ex vivo* analysis, *live vs HK*: 1.6-fold excess, ** *P* value = 0.0038 (unpaired t-test).

Table S1. Bacterial strains

The bacterial strains included in this study are listed in the table below.

Strain	Phenotype or Genotype	Source or Reference
S. aureus	Wild-type	ATCC 12600
S. aureus MRSA 1	MRSA Clinical isolate	Clinical isolate, University of Nebraska Medical Center
L. monocytogenes	Wild-type	ATCC 15313
S. epidermidis	Wild-type	ATCC 35984
K. pneumoniae	Wild-type	ATCC 13883
E. coli	Wild-type	ATCC 25922
P. aeruginosa PA01	Wild-type	ATCC 10154
A. baumannii	Wild-type	ATCC 19606
S. typhimurium	Wild-type	ATCC 29630
P. mirabilis	Wild-type	ATCC 29906
E. cloacae	Wild-type	ATCC 7256
E. faecalis	Wild-type	ATCC 19433

B. Synthetic Procedures

B.1. General:

All chemical reagents were purchased from commercial sources (Acros Organics, Alfa Aesar, AK Scientific & Sigma-Aldrich) and used without further purification unless otherwise stated. All separatory cartridges were purchased from Waters. All reactions were performed under an inert atmosphere of dry nitrogen and monitored by thin layer chromatography (TLC) on precoated (250 μm) silica gel 60 F254 aluminum sheets and visualized under a UV- 254 lamp followed by staining with potassium permanganate. Flash chromatography was performed on silica gel (60A pore size). ^{1}H, ^{13}C and ^{19}F NMR spectra were obtained on a Bruker Avance III HD 400 mHz instrument at the UCSF Nuclear Magnetic Resonance Laboratory and data were processed using MestReNova. Chemical shifts (δ) were reported in ppm relative to known solvent peaks (7.26 ppm for $CDCl_3$, 3.31 ppm for MeOD, & 4.79 ppm for D_2O). Abbreviations are as follows: s (singlet), d (doublet), t (triplet), q (quartet), m (multiplet). High resolution mass spectra (HRMS) services were provided by UC Berkeley Spectrometry Facility. The ^{18}F labeled compounds were characterized by developing the compounds in different solvent systems on silica gel TLC plates on glass followed by imaging on a radio TLC scanner (Bioscan AR2000). Analytical HPLC was performed using a Waters pump equipped with a manual Rheodyne injector (1 mL loop) and a UV detector and a RAD detector. Reversed-phase chromatography used a Phenomenex Luna C18 column stationary phase, a mobile phase of 80:20 acetonitrile (aqueous) at a flowrate of 1 mL/min, and a 254 nm observation wavelength. Chiral chromatography used a Astec Chirobiotic TAG column stationary phase, a mobile phase of 10% EtOH/90% H_2O of 1 mL/min, and a 254 nm observation wavelength. The radioactivity of the bacterial pallets and filtrate were counted on a γ counter (Hidex Automatic Gamma Counter).

B.2. Synthesis of novel compounds:

B.2.1. *tert*-butyl 3-bromo-2-((diphenylmethylene)amino)-3,3-difluoropropanoate (1)

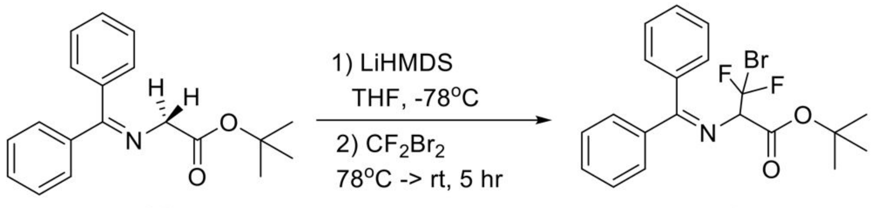

tert-butyl 3-bromo-2-((diphenylmethylene)amino)-3,3-difluoropropanoate (**1**). In a 10 mL Schlenk flask, oven dried, under N_2 gas was added (**S4**) (200 mg, 0.676 mmol), which was dissolved in anhydrous THF (2.5 mL). The mixture was cooled to -78°C with dry ice/acetone bath. LiHMDS (1.0M in THF) was added dropwise (0.8 mL, 0.811 mmol) and the reaction was left stirring at -78°C for 30 min. CF_2Br_2 (0.310 mL, 3.38 mmol) was added dropwise to the mixture and was left stirring at -78°C for 5 hours. The reaction was quenched with NH_4Cl (aq) (2mL). The organic layers were extracted using Et_2O three time (10mL). The combined organic layers were washed with brine (20 mL), then dried over Na_2SO_4. After filtration, solvent was removed using rotary evaporation. The residue was purified via column chromatography using hexanes/EtOAc (9:1) to yield compound **1** (191 mg, 78% yield):

^{1}H NMR (400 MHz, $CDCl_3$) δ 7.75 – 7.70 (m, 2H), 7.50 – 7.42 (m, 4H), 7.36 (t, J = 7.5 Hz, 2H), 7.22 – 7.18 (m, 2H), 4.50 (dd, J = 11.8, 6.7 Hz, 1H), 1.45 (s, 9H).

^{13}C NMR (100 MHz, $CDCl_3$) δ 169.11 (d), 137.13 (d), 131.20 (s), 129.37 (s), 129.26 (s), 128.74 (s), 128.16 (s), 127.65 (s), 73.69 (t), 27.83 (s).

^{19}F NMR (376 MHz, $CDCl_3$) δ -51.47 (ddd, *J* = 171.8, 160.0, 9.2 Hz).

HRMS (ESI) m/z calculated for $C_{20}H_{20}BrF_2NO_2$ (M+K) 462.02, found 462.02.

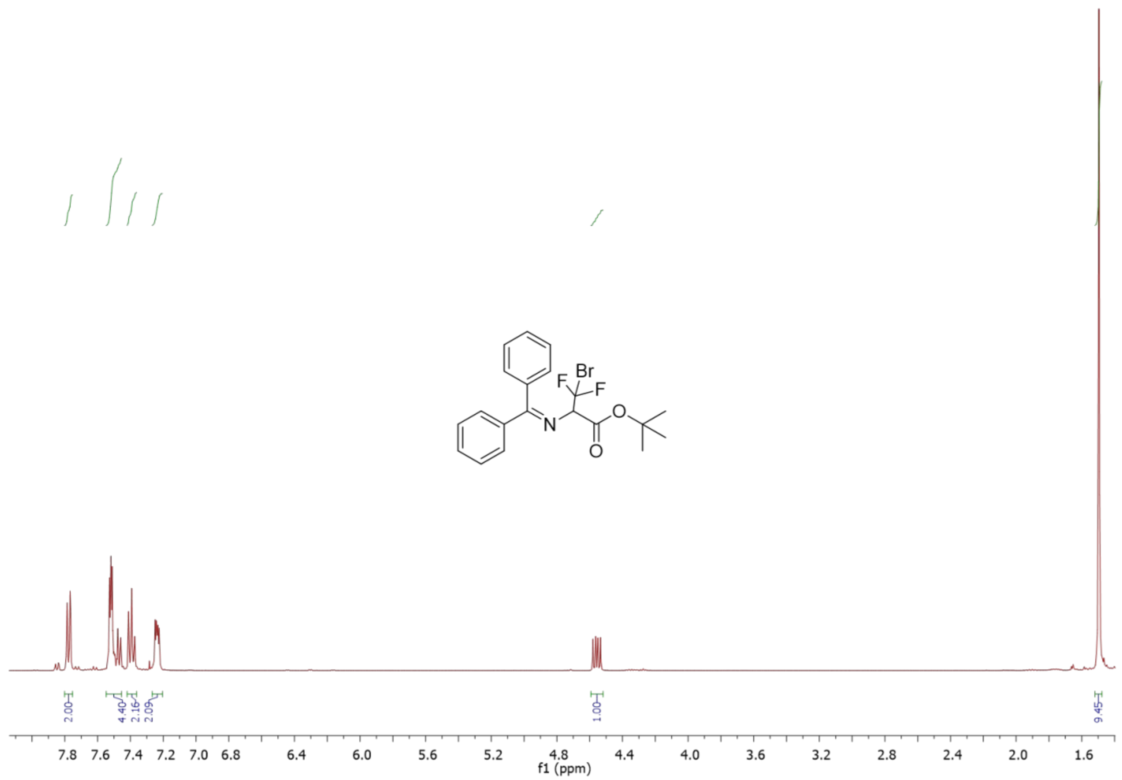

Figure B.2.1.1 ^{1}H NMR of **1** in $CDCl_3$.

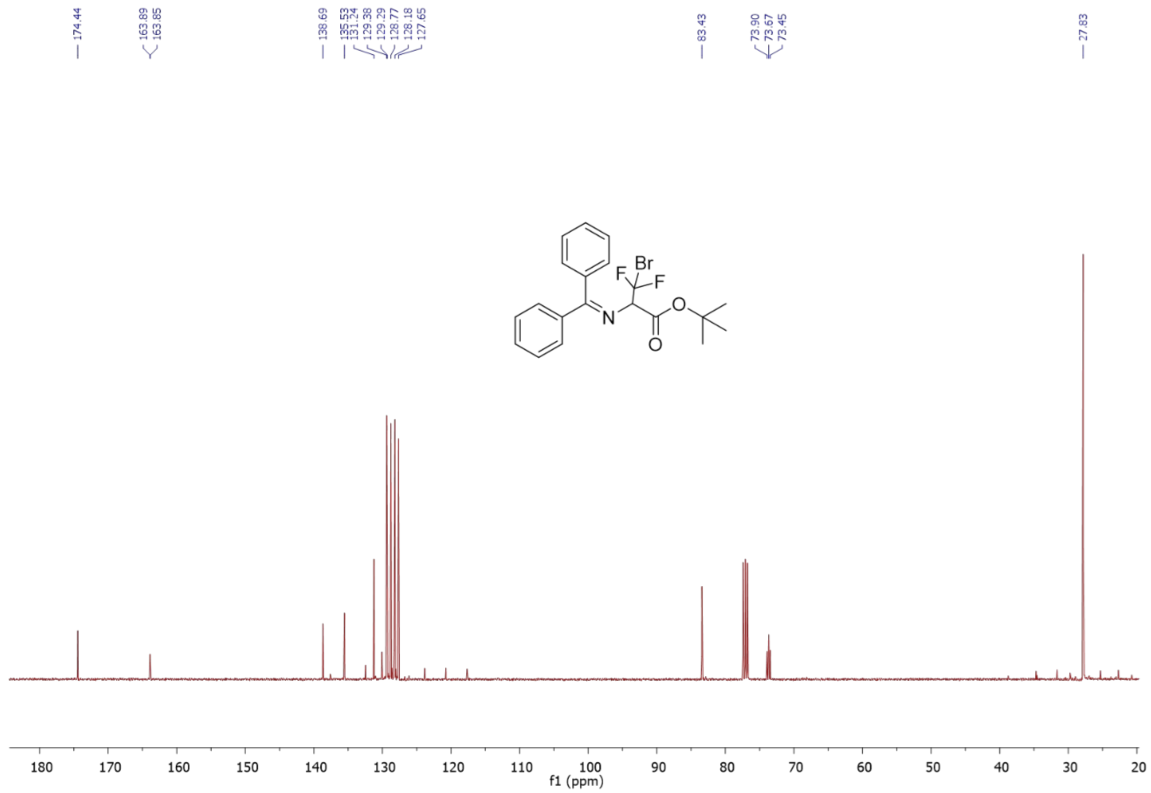

Figure B.2.1.2 ^{13}C NMR of **1** in $CDCl_3$.

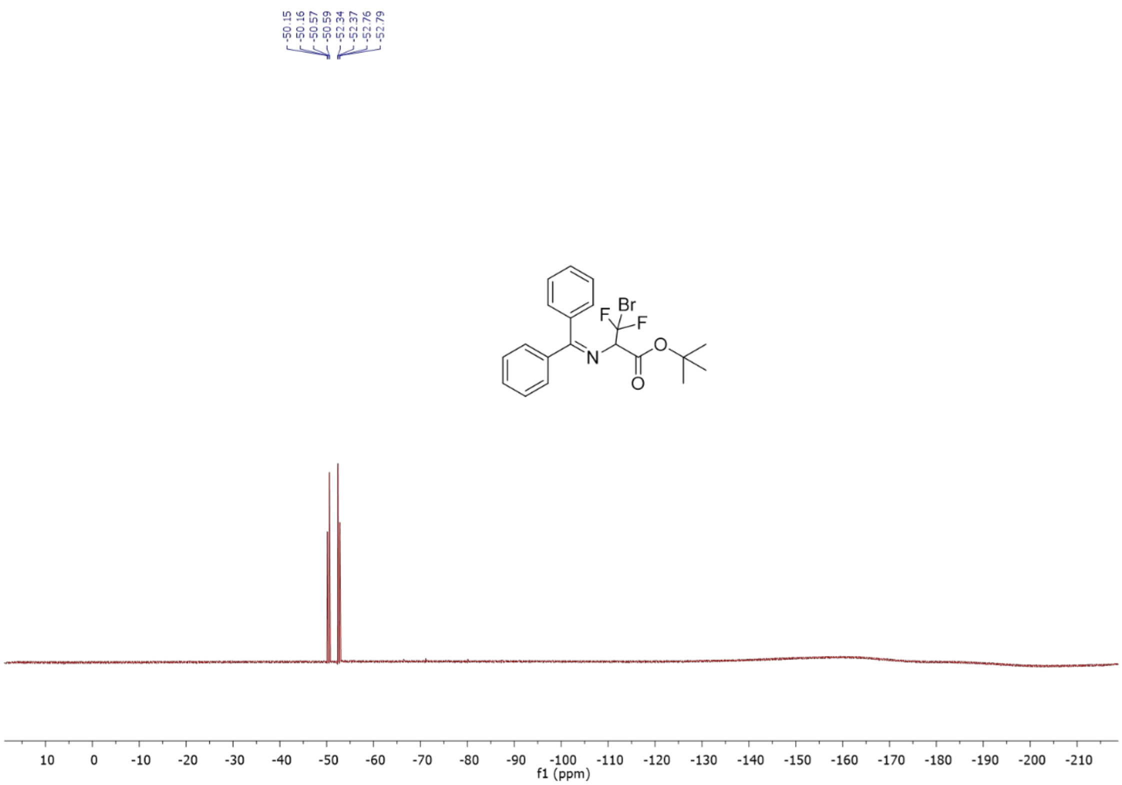

Figure B.2.1.3 ^{19}F NMR of **1** in $CDCl_3$.

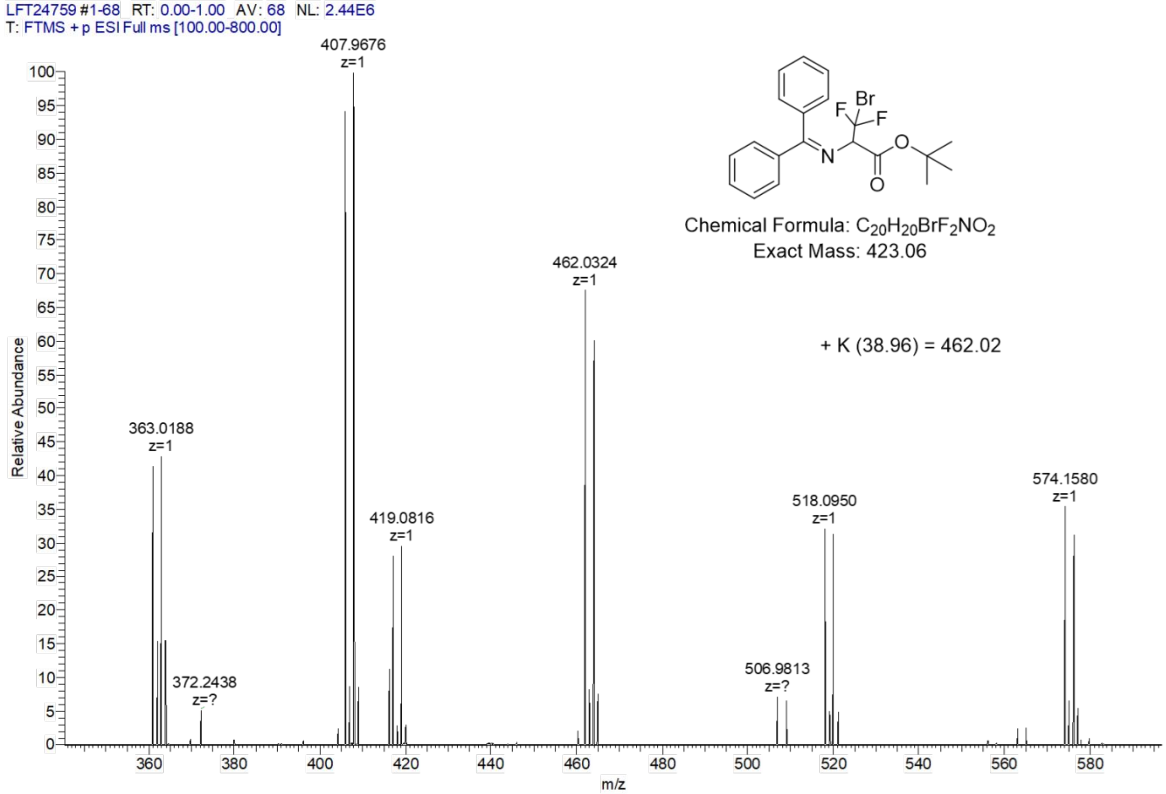

Figure B.2.1.4 HRMS of **1**.

B.2.2. ***tert-butyl 2-((diphenylmethylene)amino)-3,3,3-trifluoropropanoate*** **(3)**

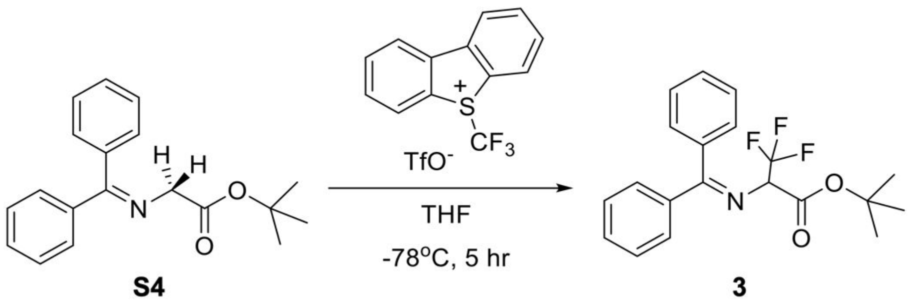

tert-butyl 2-((diphenylmethylene)amino)-3,3,3-trifluoropropanoate (**3**). In a 10 mL Schlenk flask, oven dried, under N_2 gas was added (**S4**) (100 mg, 0.336 mmol), which was dissolved in anhydrous THF (1.4 mL). The mixture was cooled to -78°C with dry ice/acetone bath. LiHMDS (1.0M in THF) was added dropwise (0.5 mL, 0.504 mmol) and the reaction was left stirring at -78°C for 30 min. Then, 5-(Trifluoromethyl)dibenzothiophenium trifluoromethanesulfonate (0.202 g, 0.504 mmol) was added dropwise to the mixture and was left stirring at -78°C for 5 hours. The reaction was quenched with NH_4Cl (aq) (2mL). The organic layers were extracted using Et_2O three time (10mL). The combined organic layers were washed with brine (20 mL), then dried over Na_2SO_4. After filtration, solvent was removed using rotary evaporation. The residue was purified via column chromatography using hexanes/EtOAc (9:1) to yield compound **3** (55 mg, 45% yield):

^{1}H NMR (400 MHz, $CDCl_3$) δ 7.76 – 7.70 (m, 2H), 7.54 – 7.44 (m, 4H), 7.37 (dd, J = 10.3, 4.6 Hz, 2H), 7.25 – 7.19 (m, 2H), 4.51 (q, J = 7.4 Hz, 1H), 1.47 (s, 9H).

^{13}C NMR (100 MHz, $CDCl_3$) δ 169.41 (d), 136.97 (d), 131.20 (s), 129.27 (s), 129.24 (s), 128.79 (s), 128.15 (s), 127.66 (s), 27.82 (s).

^{19}F NMR (376 MHz, $CDCl_3$) δ -71.09 (d, *J* = 7.4 Hz).

HRMS (ESI) m/z calculated for $C_{20}H_{20}F_3NO_2$ (M+H) 363.14, found 364.15.

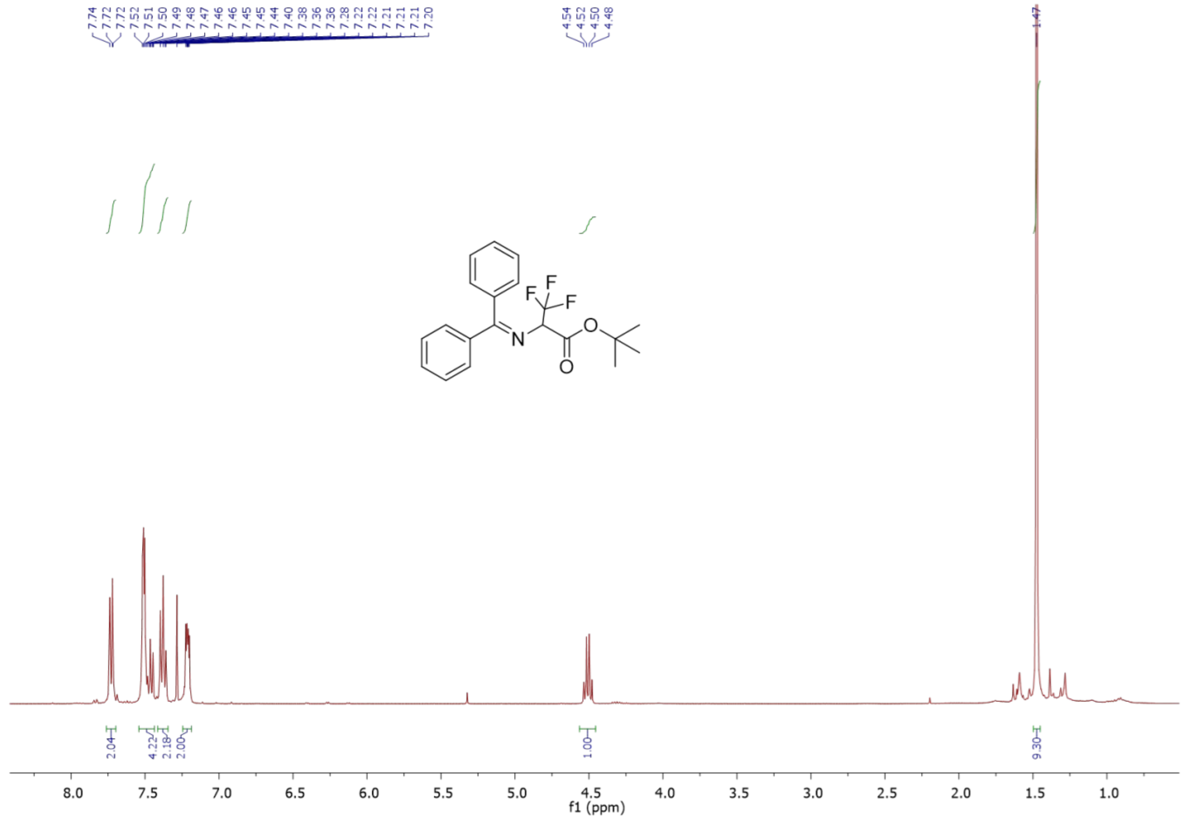

Figure B.2.2.1 ^{1}H NMR of **3** in $CDCl_3$.

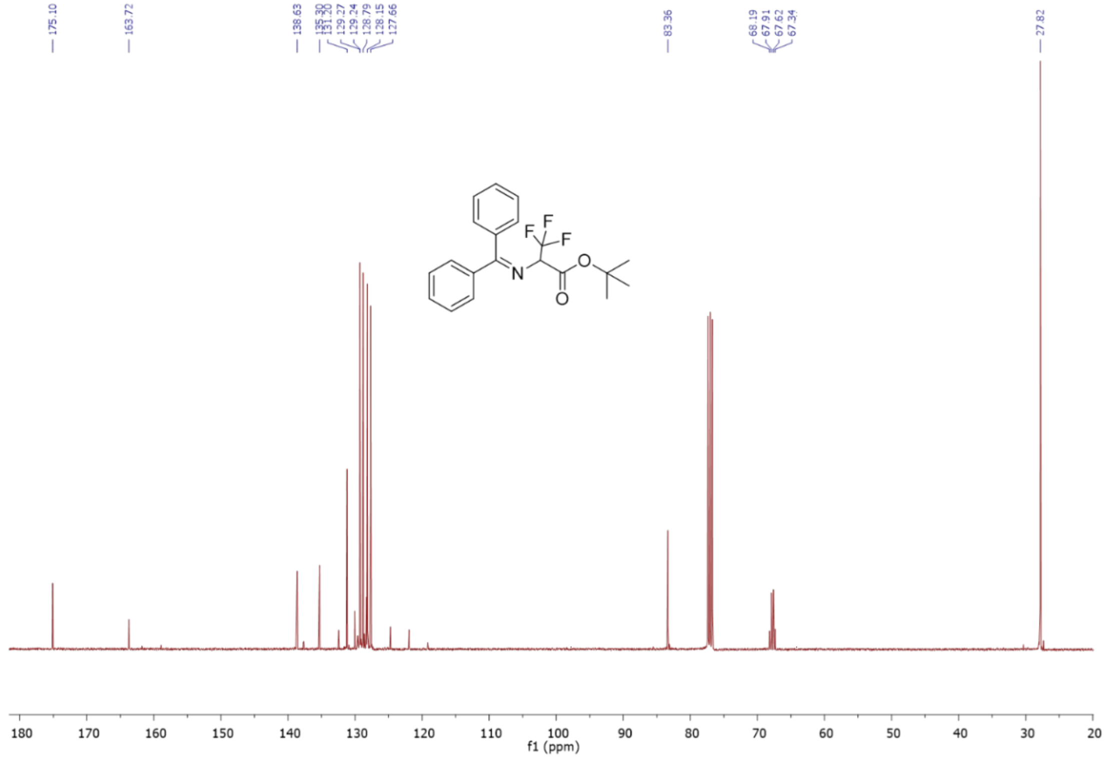

Figure B.2.2.2 ^{13}C NMR of **3** in $CDCl_3$.

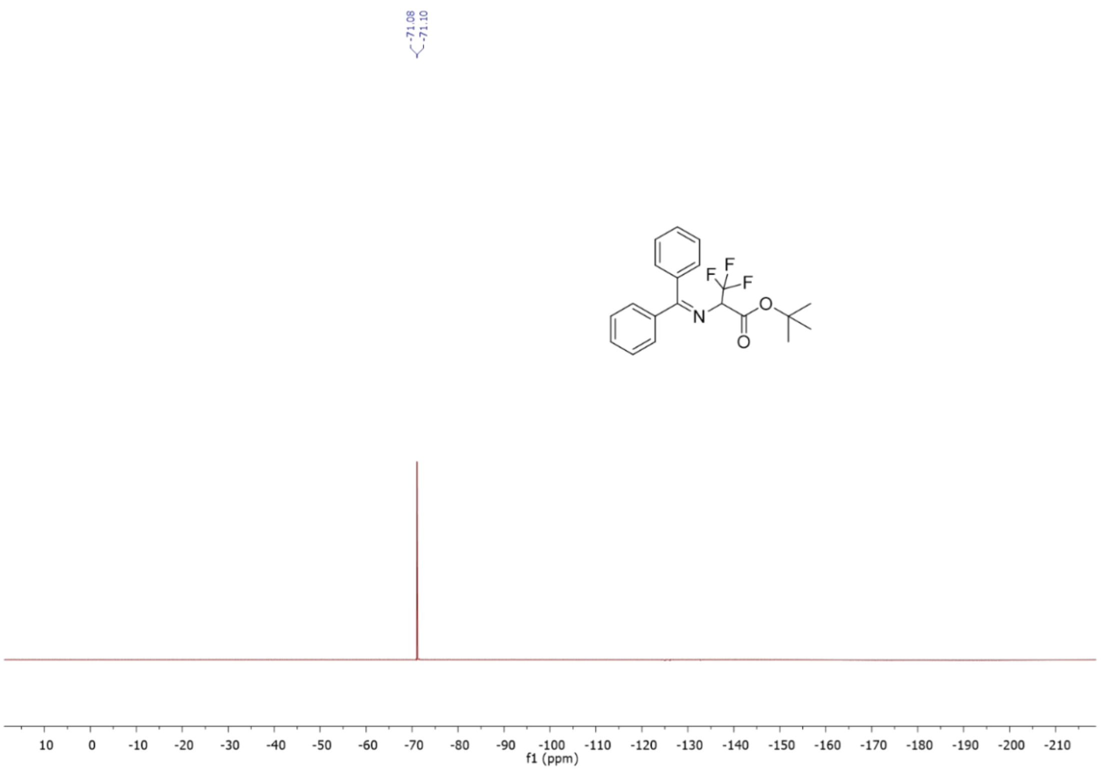

Figure B.2.2.3 ^{19}F NMR of **3** in $CDCl_3$.

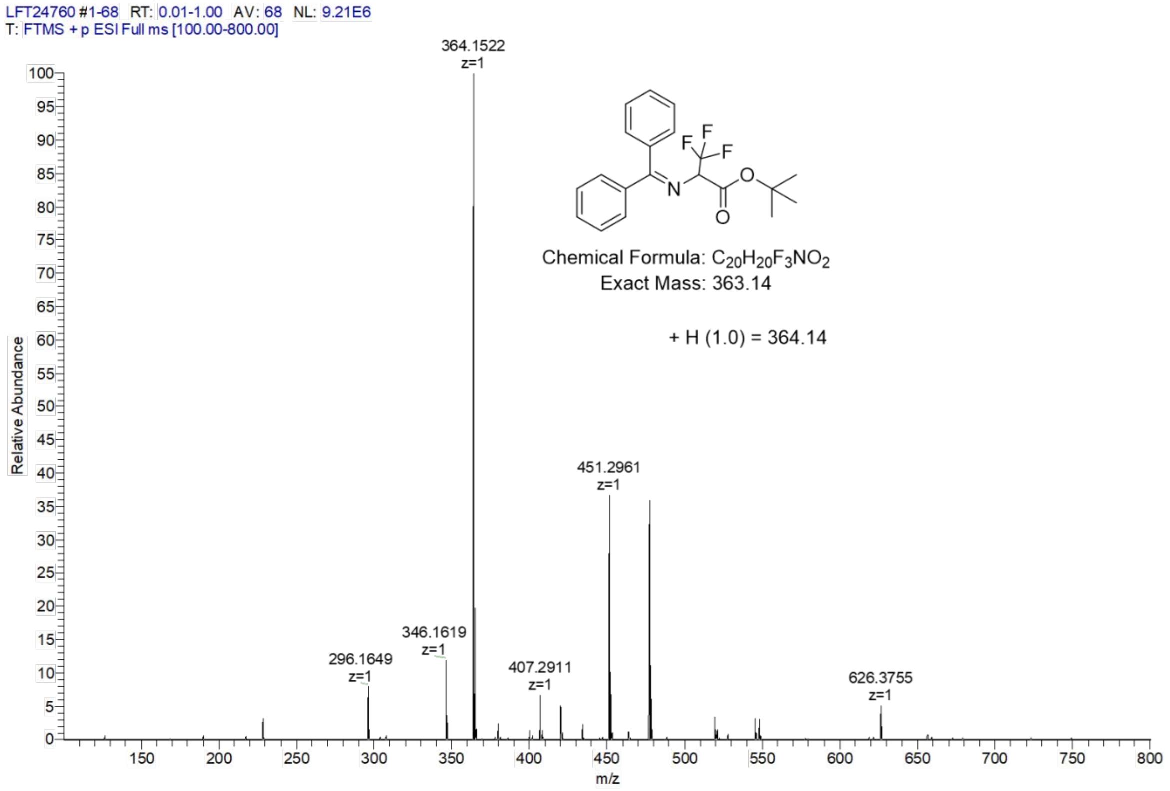

Figure B.2.2.4 HRMS of **3**.

C. Radiochemistry

C.1. Radiochemical procedure for [^{18}F]KF/K_{222} generation

[^{18}F]fluoride was obtained from the University of California, San Francisco Radiopharmaceutical Facility, where it was generated by proton irradiation of enriched [^{18}O]H_2O by a PET trace 18 MeV cyclotron (GE Healthcare, Buckinghamshire U.K.). ^{18}F ion (25 millicuries) in [^{18}O]H_2O was passed through a QMA anion exchange column, conditioned with 1M K_2CO_3 (2 mL) and water (3 mL). ^{18}F was eluted with K_2CO_3 (2 mg) and kryptofix K_{222} (12 mg) dissolved in a 1 mL water-acetonitrile (1-1 v/v) mix. Next, the solvent was dried down under vacuum and nitrogen at 110 °C.

C.2. Radiosynthesis of D-[^{18}F]-CF_3-alanine:

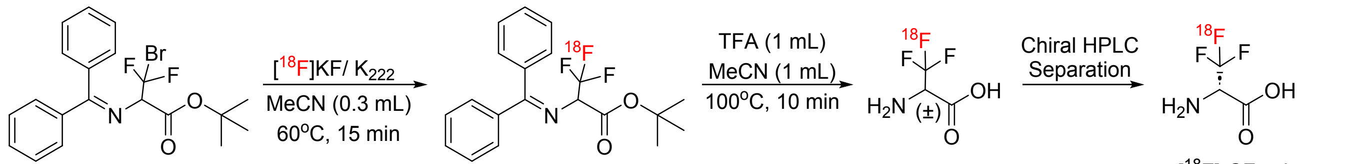

D-[^{18}F]-CF_3-ala

RCY = 36.3 ± 5.1%, RCP > 99%

%*ee* = 90.5 ± 1.7%, n = 11

A_m = 0.03 ± 0.004 GBq/μmol

In a reaction vessel, CF_2Br precursor **1** (16 mg,0.038 mmol) was dissolved in 0.3 mL of MeCN and added to the dried down ^{18}F-K_2CO_3-K_{222} mix and heated to 60 °C for 15 min. RadioTLC (1:9 hexane:ethyl acetate) and analytical HPLCs showed formation of ^{18}F labeled compound **2**. Average radiochemical conversion **2**: (86.3 ± 5.1)% (N=11). A sample was taken for analysis on a Phenomenex Luna C18 column (acetonitrile/H_2O, 80/20, *v/v*) with a retention time (t_R) of 13 min for [^{18}F]CF_3 intermediate **2**. The reaction mixture was purified via semi prep HPLC using a Phenomenex Luna C18 column (4mL/min, acetonitrile/H_2O, 80/20, *v/v*). The fractions containing **2** at retention time (t_R) of 17 min were collected, then diluted in 40 mL of dH_2O. The diluted mixture was passed through a C18 plus short Waters Sep Pak, preconditioned with ethanol (5 mL) and dH_2O (10 mL). The cartridge was then eluted with 1 mL of MeCN. To the eluted fraction was added 1 mL of TFA and heated to 100°C for 10 min. A sample was taken for analysis on a Phenomenex Luna C18 column (acetonitrile/H_2O, 80/20, *v/v*) to confirm the radiosynthesis of (±)-[^{18}F]-CF_3-alanine. The reaction mixture was concentrated and diluted with 1mL of MeCN x 3 to remove all the TFA. The residue was diluted with H_2O then purified via semi prep HPLC (Astec Chirobiotic TAG, 250 X 10 mm) using (3 mL/min, EtOH/H_2O, 15/85, *v/v*). The fractions containing

D[^{18}F]-CF_3-alanine at retention time (t_R) of 5 min were collected, then diluted in saline. RCY = 36.3 ± 5.1% (decay-corrected), RCP > 99%, %ee = 90.5 ± 1.7%, N =11, Am = 0.036 ± 0.004 GBq/μmol, synthesis time = 95 min and EOS RCY = 19.9± 2.8% (non-decay corrected).

C.2.1. Radio HPLC and TLC Analysis of 1st Step

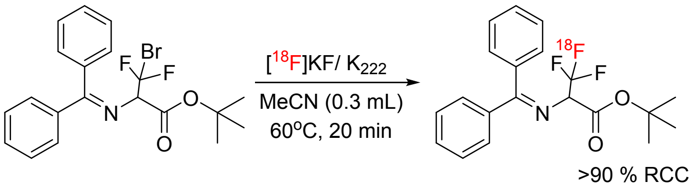

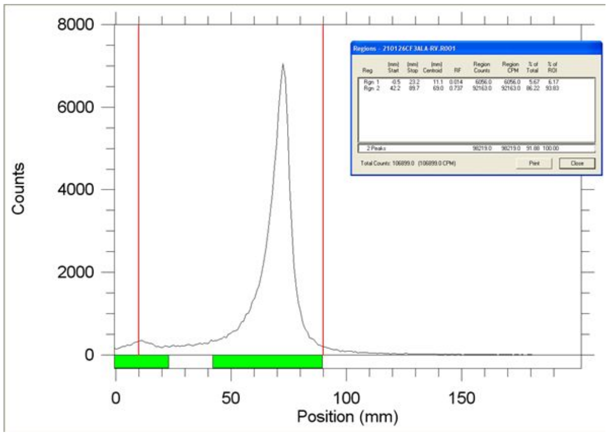

Figure C.2.1.1. Radio TLC silica gel step 1

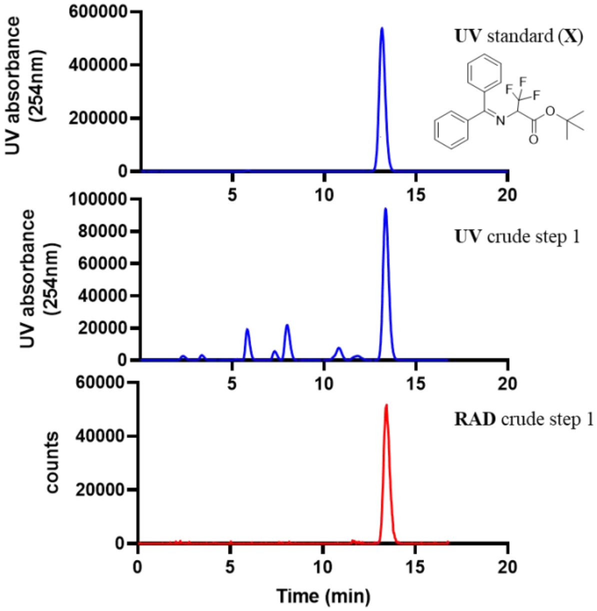

Figure C.2.1.2. Reverse Phase Analytical HPLC Step 1, the radiolabeled [^{18}F]-CF_3 intermediate **2** elutes at 13 min (red).

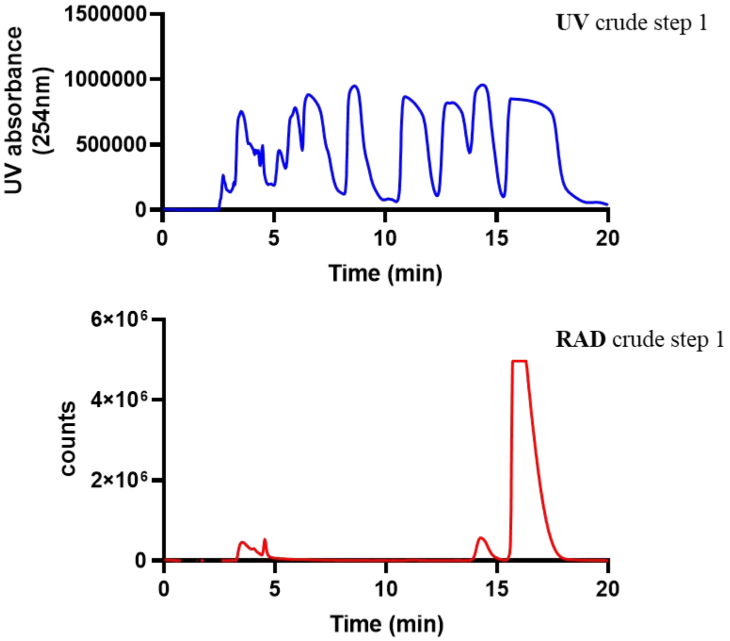

Figure C.2.1.3. Reverse Phase Semi Prep HPLC for purification after first step with radiolabeled [^{18}F]-CF_3- intermediate **2** elutes at 17 min (red).

C.2.2. Radio HPLC and TLC Analysis of 2nd Step

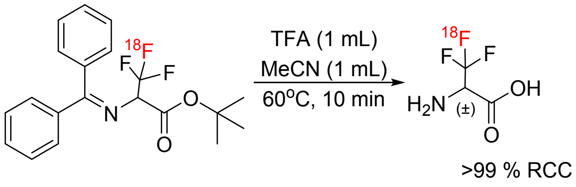

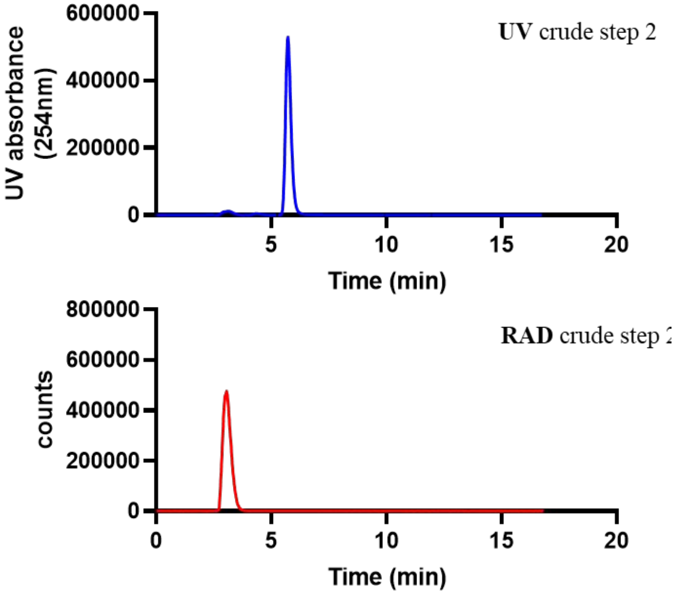

Figure C.2.2.2. Reverse Phase Analytical HPLC Step 2

C.2.3. Chiral HPLC Separation

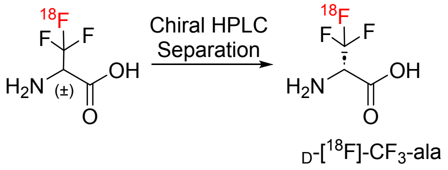

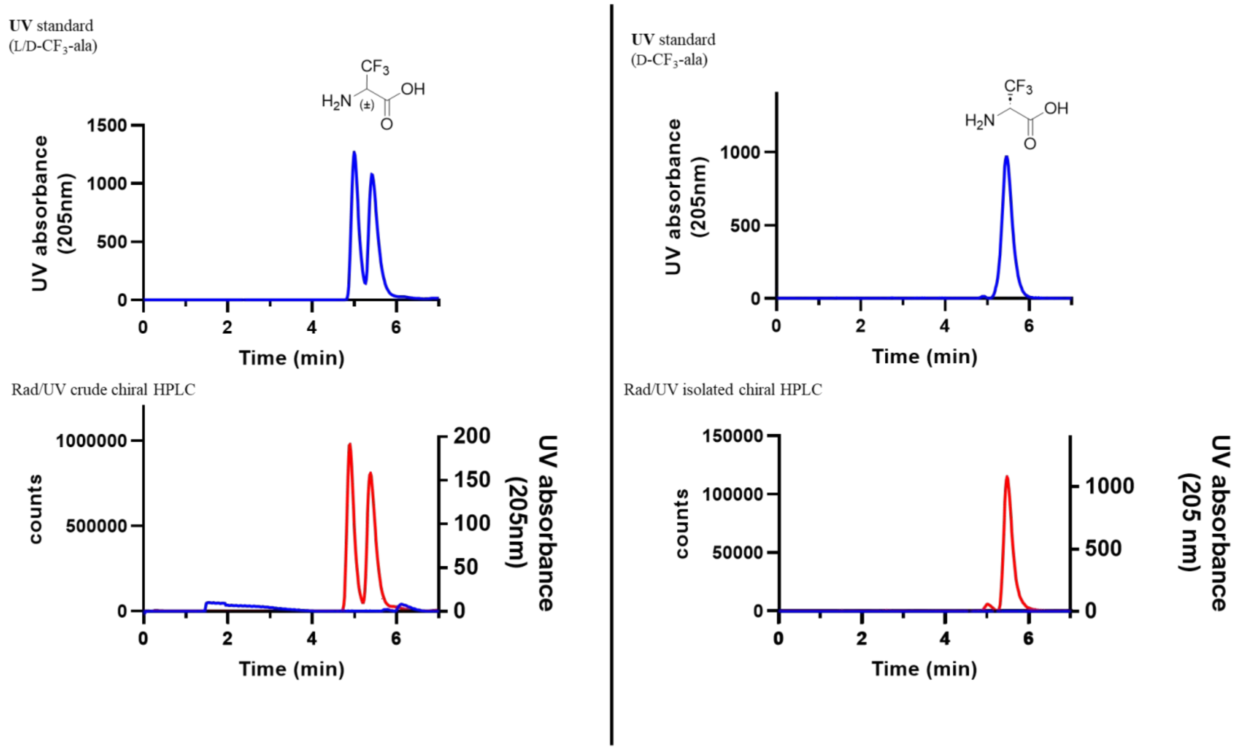

Figure C.2.3.1. Chiral Semi Prep and analytical HPLC (A) Chiral HPLC of radiolabeled racemic [18F]-CF_3-alanine product after second synthetic step. The deprotected radiolabeled [18F]-CF3-alanine elutes at 5.0 min for the L-enantiomer and 5.5 min for the D-enantiomer. (B) Chiral HPLC of final radiolabeled D-[18F]-CF_3-alanine (RT 5.5 min).

D. References

(1) Parker, M. F. L.; Luu, J. M.; Schulte, B.; Huynh, T. L.; Stewart, M. N.; Sriram, R.; Yu, M. A.; Jivan, S.; Turnbaugh, P. J.; Flavell, R. R.; Rosenberg, O. S.; Ohliger, M. A.; Wilson, D. M. Sensing Living Bacteria in Vivo Using D-Alanine-Derived 11C Radiotracers. *ACS Cent. Sci.* **2020**, *6* (2), 155–165. DOI: 10.1021/acscentsci.9b00743.
